# The Human Bcl-2 Family Member Bcl-rambo Localizes to Mitochondria and Induces Apoptosis and Morphological Aberrations in *Drosophila*

**DOI:** 10.1371/journal.pone.0157823

**Published:** 2016-06-27

**Authors:** Mako Nakazawa, Hisanori Matsubara, Yuka Matsushita, Megumi Watanabe, Nicole Vo, Hideki Yoshida, Masamitsu Yamaguchi, Takao Kataoka

**Affiliations:** 1 Department of Applied Biology, Kyoto Institute of Technology, Matsugasaki, Sakyo-ku, Kyoto 606–8585, Japan; 2 The Center for Advanced Insect Research Promotion (CAIRP), Kyoto Institute of Technology, Matsugasaki, Sakyo-ku, Kyoto 606–8585, Japan; The University of Texas MD Anderson Cancer Center, UNITED STATES

## Abstract

Bcl-2 family proteins play a central role in regulating apoptosis. We previously reported that human Bcl-rambo, also termed BCL2L13, localized to mitochondria and induced apoptosis when overexpressed in human embryonic kidney 293T cells. However, the physiological function of Bcl-rambo currently remains unclear. In the present study, human Bcl-rambo was ectopically expressed in *Drosophila melanogaster*. Bcl-rambo mainly localized to the mitochondria of *Drosophila* Schneider 2 (S2) cells. The overexpression of Bcl-rambo, but not Bcl-rambo lacking a C-terminal transmembrane domain, induced apoptosis in S2 cells. Moreover, the ectopic expression of Bcl-rambo by a GAL4-UAS system induced aberrant morphological changes characterized by atrophied wing, split thorax, and rough eye phenotypes. Bcl-rambo induced the activation of effector caspases in eye imaginal discs. The rough eye phenotype induced by Bcl-rambo was partly rescued by the co-expression of p35, Diap1, and Diap2. By using this *Drosophila* model, we showed that human Bcl-rambo interacted genetically with *Drosophila* homologues of adenine nucleotide translocators and the autophagy-related 8 protein. The results of the present study demonstrated that human Bcl-rambo localized to mitochondria and at least regulated an apoptosis signaling pathway in *Drosophila*.

## Introduction

Programmed cell death plays an essential role in the development and maintenance of tissue homeostasis in animals [1]. Apoptosis is one type of programmed cell death that is mainly regulated by a family of cysteinyl aspartate-specific proteinases (caspases) [2]. Caspases have been classified as initiator caspases (e.g., caspase-8, -9, and -10) and effector caspases (e.g., caspase-3, -6, and -7) [3]. Upon apoptotic stimuli, initiator caspases are activated in platforms referred to as an apoptosome or death-inducing signaling complex, and then cleave effector caspases into their active forms [4,5]. Active effector caspases have been shown to mediate the proteolytic cleavage of many target proteins, leading to the execution of apoptosis [6].

Mitochondria are central in the regulation of the intrinsic apoptosis pathway, which is regulated primarily by Bcl-2 family proteins [7]. Bcl-2 family proteins possess at least one of four Bcl-2 homology (BH) domains, and have been classified into three groups: anti-apoptotic (pro-survival) proteins (e.g., Bcl-2 and Bcl-x_L_), pro-apoptotic proteins (e.g., Bax and Bak), and BH3-only proteins (e.g., Bad, Bid, and Bim) [8]. Anti-apoptotic proteins interact with pro-apoptotic multidomain proteins, suppressing their activity, whereas BH3-only proteins either interact with anti-apoptotic proteins to inhibit their function or with pro-apoptotic multidomain proteins to stimulate their function [9]. Bcl-2 family proteins have been shown to regulate mitochondrial outer membrane permeabilization, which allows the release of pro-apoptotic proteins, including cytochrome *c* from the intermembrane space to the cytoplasm [10,11]. Released cytochrome *c* induces the formation of an apoptosome, thereby promoting the activation of the initiator caspase-9 [4].

**Fig 1:**
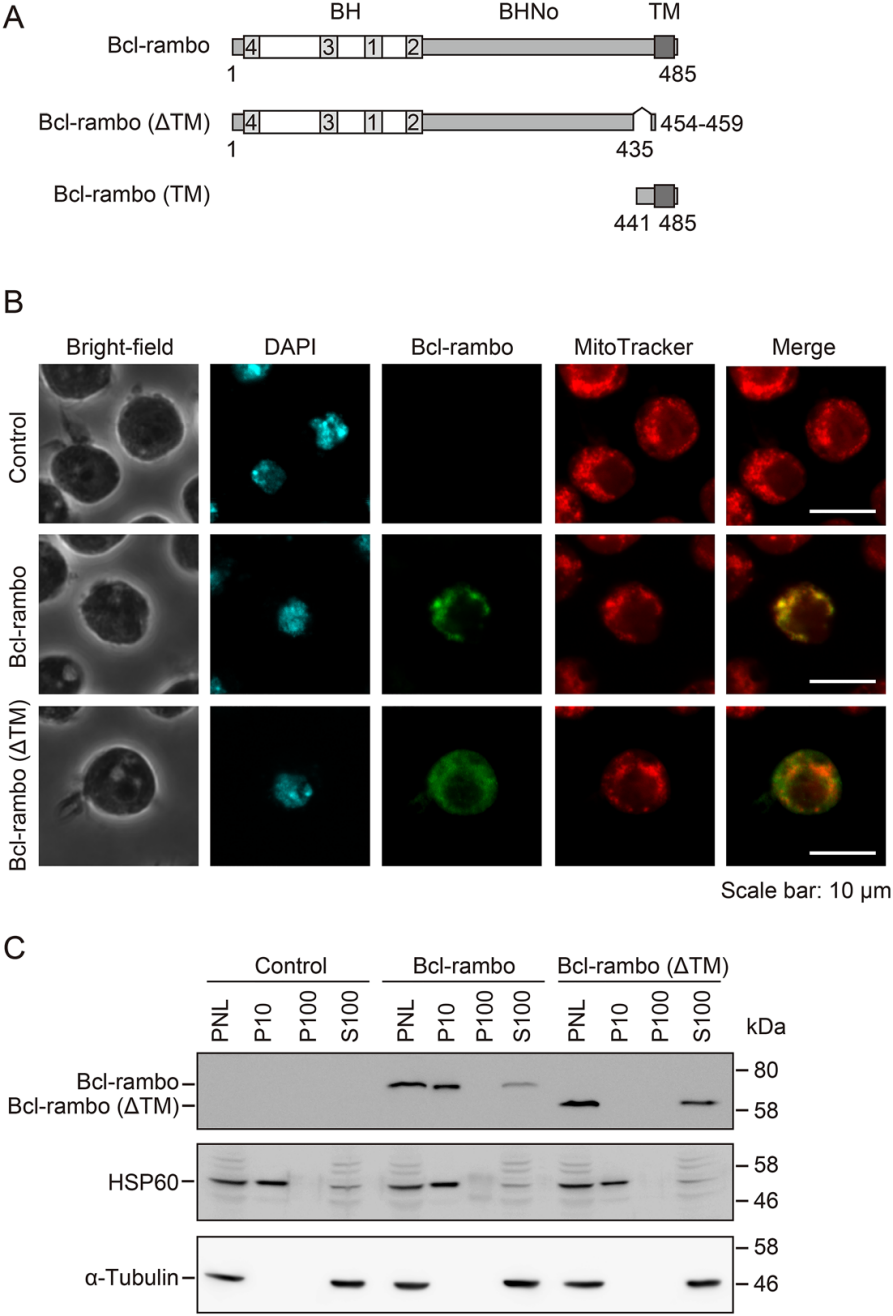
Bcl-rambo localized to mitochondria in *Drosophila* S2 cells. (A) Structures of human Bcl-rambo and its mutants. (B) S2 cells were transfected with pMT-V5-His A, pMT-V5-His A/*Bcl-rambo*, or pMT-V5-His A/*Bcl-rambo (ΔTM)* and then incubated in the presence of CuSO_4_ (500 μM) and Z-VAD-fmk (20 μM) for 24 h. S2 cells were stained for Bcl-rambo (green) and with DAPI (blue) and MitoTracker^®^ Red (red). The stained cells in at least five different fields were observed by confocal laser scanning microscopy. Optical sections containing single transfected cells are shown. Data were representative of two independent experiments. Scale bars indicate 10 μm. (C) S2 cells were transfected with pMT-V5-His A, pMT-V5-His A/*Bcl-rambo*, or pMT-V5-His A/*Bcl-rambo (ΔTM)* and incubated in the presence of CuSO_4_ (500 μM) for 24 h. S2 cells were homogenized and separated into PNL, P10, P100, and S100 fractions. The fractions were analyzed by Western blotting using anti-Bcl-rambo, anti-HSP60, and anti-α-tubulin antibodies. Data were representative of three independent experiments.

We previously identified the widely expressed Bcl-2 family protein Bcl-rambo, also termed BCL2L13 (Fig 1A) [12], and showed that it was composed of four N-terminal BH domains (i.e., BH1, BH2, BH3, and BH4), a unique 250 amino acid extension termed the BHNo domain, and a C-terminal transmembrane domain (TM) [12]. Bcl-rambo localized to mitochondria and induced apoptosis when it was overexpressed in human embryonic kidney (HEK) 293T cells [12]. Bcl-rambo was previously shown to be strongly expressed in various cancer cells, i.e., childhood acute lymphoblastic leukemia, liposarcoma, gastric cancer, and glioblastoma [13–17]. It was also found to be constitutively expressed throughout human early embryonic development [18]. Moreover, it has been reported that Bcl-rambo regulates apoptosis [17,19–22] or induces mitochondrial fragmentation and mitophagy [23]. Although Bcl-rambo has been suggested to either positively or negatively regulate apoptosis, its physiological function remains unclear.

*Drosophila melanogaster* is commonly used as a model organism and is a powerful screening tool for investigating genetic interactions. Mammals and flies share some common features for apoptosis signaling pathways [24–26]. *Drosophila* possesses apoptosis regulators, such as Bcl-2 family proteins, caspases, and inhibitor of apoptosis (IAP) proteins [27–29]. In the present study, we ectopically expressed human Bcl-rambo using a GAL4-UAS expression system in *Drosophila*. The results obtained showed that Bcl-rambo localized to mitochondria. We also demonstrated that the ectopic expression of Bcl-rambo in transgenic flies induced apoptosis and caused aberrant morphological changes, including a rough eye phenotype.

## Materials and Methods

### Cells

*Drosophila* Schneider 2 (S2) cells were grown in Schneider's *Drosophila* medium (Life Technologies, Grand Island, NY, USA) containing heat-inactivated fetal calf serum (Nichirei Bioscience, Tokyo, Japan) and penicillin-streptomycin mixed solution (Nacalai Tesque, Kyoto, Japan) at 25°C. HEK 293T cells were maintained in DMEM medium (Life Technologies) containing heat-inactivated fetal calf serum and penicillin-streptomycin mixed solution at 37°C.

### Plasmids

Human Bcl-rambo (1–485) (Fig 1A) was described previously [12]. Bcl-rambo (1–435, 454–459) designated Bcl-rambo (ΔTM), Bcl-rambo (441–485) designated Bcl-rambo (TM), Bcl-rambo (1–441), and Bcl-rambo (1–459) were generated by PCR amplification (Fig 1A and panel A in S1 Fig). Full-length Bcl-rambo, Bcl-rambo (ΔTM), Bcl-rambo (TM), Bcl-rambo (1–441), Bcl-rambo (1–459), and DsRed-monomer (TAKARA BIO, Shiga, Japan) were inserted into pUAST and/or pMT/V5-His A expression vectors. The genes encoding Drob-1/Debcl and Buffy were generated by PCR amplification using a cDNA library prepared from *Drosophila* early embryos. Full-length Drob-1/Debcl and Buffy were inserted into pCR3-based expression vectors containing N-terminal FLAG or VSV tags. FLAG-tagged Drob-1 was inserted into pMT/V5-His A expression vectors.

### Establishment of transgenic flies

pUAST-*Bcl-rambo* and pUAST-*Bcl-rambo (ΔTM)* were injected into embryos to obtain stable transformant lines carrying UAS-*Bcl-rambo* and UAS-*Bcl-rambo (ΔTM)*. P element-mediated germline transformation was accomplished as described [30]. F1 transformants were selected on the basis of white eye color rescue. Eight and six independent lines were established for UAS-*Bcl-rambo* and UAS-*Bcl-rambo (ΔTM)*, respectively.

### Fly stocks

Fly stocks were maintained at 25°C by standard food (0.7% agar, 5% glucose, and 7% dry yeast). The UAS-*Bcl-rambo* and UAS-*Bcl-rambo (ΔTM)* fly lines were established in the present study. The fly lines carrying *glass multiple reporter* (*GMR*)-*GAL4* were described previously [31]. The *engrailed* (*en*)-*GAL4* driver fly line was kindly provided by Dr. Nicholas Dyson (Massachusetts General Hospital, Boston, MA, USA). UAS-*GFP*, UAS-*p35*, UAS-*Diap1*, *GMR*-*Diap2*, *pannier* (*pnr*)-*GAL4*, *decapentaplegic* (*dpp*)-*GAL4*, *salivary gland* (*sg*)-*GAL4*, *Drob-1*^*E26*^, *Drob-1*^*W105*^, *Drob-1*^*E26*^ *Buffy*^*H37*^, *Drob-1*^*W105*^ *Buffy*^*H37*^, *Buffy*^*H37*^, *sesB*^*org*^, *Ant2*^*G0247*^ *sesB*^*G0247*^, *schlank*^*G0061*^, and *Atg8a*^*EP362*^ fly lines were obtained from the Bloomington *Drosophila* stock center (Bloomington, IN, USA) and the *Drosophila* Genetic Resource Center (Kyoto, Japan). The *GAL4* driver fly lines were crossed with the UAS fly lines, developed, and then reared at 28°C.

### Antibodies

Antibodies reactive to Bcl-rambo (Rocky-1; Santa Cruz Biotechnology, Santa Cruz, CA, USA), cytochrome *c* (7H8.2C12; BD Biosciences, San Jose, CA, USA), FLAG (1E6, Wako Pure Chemical Industries, Osaka, Japan), HSP60 (insect) (Enzo Life Science, Farmingdale, NY, USA), α-tubulin (DM1A; Sigma-Aldrich, St. Louis, MI, USA), and VSV-G (P5D4; Santa Cruz Biotechnology) were commercially obtained. Antibodies reactive to Cut (2B10), Discs large (4F3), Elav (7E8A10), and Prospero (MR1A) were obtained from the Developmental Studies Hybridoma Bank (Iowa City, IA, USA). Secondary antibodies conjugated to horseradish peroxidase (HRP) were purchased from Jackson ImmunoResearch (West Grove, PA, USA).

### Transfection

S2 cells were transfected with expression vectors by HilyMax transfection reagent (Dojindo, Tokyo, Japan) or siLentfect^™^ lipid reagent (Bio-Rad Laboratories, Hercules, CA, USA).

### Immunostaining

S2 cells were incubated with MitoTracker^®^ Red CMXRos (Lonza, Basel, Switzerland) for 30 min prior to fixation. The caspase inhibitor Z-Val-Ala-Asp(OMe)-fluoromethylketone (Z-VAD-fmk; Peptide Institute, Osaka, Japan) was used to prevent cell death. Tissues were dissected in phosphate-buffered saline (PBS). S2 cells and tissues were fixed with 4% paraformaldehyde (PFA)–PBS and treated with 0.1% and 0.3% Triton X-100–PBS, respectively. The permeabilized cells were blocked with normal goat serum and incubated with primary antibodies and secondary antibodies conjugated with Alexa Fluor 488 (Life Technologies). S2 cells and tissues were then stained with Hoechst 33342 and 4’,6-diamidino-2-phenylindole (DAPI) at final concentrations of 7.5 μM and 1.5 μg/ml, respectively. Samples were analyzed by the confocal laser scanning microscope FV10i (Olympus, Tokyo, Japan).

### Subcellular fractionation

S2 cells were transfected with pMT/V5-His A expression vectors encoding Bcl-rambo and Bcl-rambo (ΔTM), and incubated with CuSO_4_ (500 μM) for 24 h. The cells were washed with PBS and disrupted by a dounce homogenizer (15 strokes) in Hepes-sucrose buffer containing 250 mM sucrose, 10 mM Hepes-KOH (pH 7.4), 1 mM EDTA, 1 mM EGTA, and the protease inhibitor cocktail Complete^™^ (Roche Diagnostics, Mannheim, Germany). Homogenates were centrifuged (500 x *g*, 10 min) to remove nuclei and unbroken cells. Supernatants were collected as post nuclear lysates (PNL) and further centrifuged (10,000 x *g*, 15 min) for separation into precipitates (P10 fraction) and supernatants. The supernatants were centrifuged (100,000 x *g*, 60 min) and separated into precipitates (P100 fraction) and supernatants (S100 fraction). Alternatively, S2 cells were washed with PBS and treated with digitonin lysis buffer (10 mM Hepes-KOH (pH 7.2), 100 μM digitonin, 250 mM sucrose, 50 mM NaCl, 5 mM EGTA, 2 mM MgCl_2_, 1 mM dithiothreitol, Complete^™^) on ice for 15 min. Cell lysates were centrifuged (15,300 x *g*, 5 min) to separate supernatants as cytosolic fractions. Precipitates were treated with Triton X-100 lysis buffer (50 mM Tris-HCl (pH 7.4), 1% Triton X-100, 2 mM dithiothreitol, 2 mM sodium orthovanadate, and Complete^™^) on ice for 15 min and centrifuged (15,300 x *g*, 5min) to remove insoluble materials. Supernatants were collected as organelle fractions containing mitochondria.

### Western blotting

Subcellular fractions were prepared as described above. Salivary glands were taken from third instar larva and heated at 95°C for 2 min in 0.11 mM Tris-HCl (pH 7.5) and Complete^™^. They were homogenized with a dounce homogenizer in the presence of SDS sample buffer. After centrifugation (10, 000 x *g*, 10 min), supernatants were collected. Protein samples were separated by SDS-PAGE and transferred onto nitrocellulose membranes (Wako Pure Chemical Industries, Osaka, Japan). The membranes were incubated with primary antibodies and then HRP-conjugated secondary antibodies. Protein bands were visualized using ECL Western Blotting Detection Reagents (GE Healthcare, Piscataway, NJ, USA) and analyzed by ImageQuant LAS 4000 mini (GE Healthcare).

### Apoptosis assay

S2 cells were stained with Hoechst 33342 (7.5 μM). Nuclear morphology was observed under a fluorescence light microscope (Axiovert 200M, Carl Zeiss, Jena, Germany). Apoptotic cells (%) were calculated as (condensed and/or fragmented nuclei / total nuclei) x 100. In parallel with the apoptosis assay, transfection efficiency was evaluated by transfection with expression vectors encoding DsRed-monomer.

### Light microscopy and scanning electron microscopy (SEM)

Adult wings were dehydrated with isopropanol and mounted in Hoyer’s medium. Samples were observed under a light microscope SZX12 (Olympus) or SEM VE-7800 (Keyence, Osaka, Japan) in the low vacuum mode. The phenotypes of the eye and thorax from at least four adult female flies (3 to 5 d old) were observed. The phenotypes of the wings from 4–5 adult female flies (3 to 5 d old) were observed. In these experiments, no significant variations were observed in eye, thorax, and wing phenotypes among the individuals. The L3-L4 area of the wings was quantified by Image J software. The mean of the L3-L4 area was calculated from 4–5 flies and normalized by the mean of the control L3-L4 area. The L3-L4 area (%) was calculated as the mean ± S.E. of three independent experiments. The brightness and contrast of all images taken by the light microscope were adjusted uniformly using Microsoft Office Picture Manager, which was applied equally to all images in each figure.

### Caspase assay

Third instar larvae were dissected in PBS, and imaginal discs were suspended in Grace’s insect medium in the presence of Cellevent^™^ Caspase-3/7 Green Detection Reagent (Life Technologies) at the final concentration of 5 μM for 1 h at 37°C. Eye imaginal discs were fixed with 4% PFA–PBS and permeabilized with 0.5% Triton X-100–PBS, followed by staining with Hoechst 33342 (5 μM). Samples were analyzed by confocal laser scanning microscopy.

### Immunoprecipitation

HEK293T cells were transfected with expression vectors by the calcium phosphate method and incubated for in the presence of Z-VAD-fmk (20 μM) for 16 h. Cells were solubilized in Nonidet P-40 lysis buffer (20 mM Tris-HCl (pH 7.4), 150 mM NaCl, 0.5% Nonidet P-40, 10% glycerol, and 2 mM sodium vanadate, Complete^™^). Postnuclear lysates were precleared with Sepharose 6B for 1 h and then immunoprecipitated with anti-FLAG M2 affinity gels (Sigma-Aldrich) for 3 h. The immunoprecipitates were washed several times with Nonidet P-40 lysis buffer and analyzed by Western blotting.

### Statistical analysis

Statistical analyses were performed using an analysis of variance followed by the Tukey’s test for multiple comparisons.

## Results

### Bcl-rambo localized to mitochondria in *Drosophila* S2 cells

Bcl-2 family proteins regulate the intrinsic apoptosis pathway via mitochondria. In HEK293T cells, full-length Bcl-rambo (1–485), but not Bcl-rambo lacking the C-terminal TM (1–459), localized to mitochondria [12]. Bcl-rambo, Bcl-rambo (ΔTM), Bcl-rambo (1–441), and Bcl-rambo (1–459) were transiently expressed in *Drosophila* S2 cells in order to examine whether the C-terminal TM of Bcl-rambo was necessary for localization to mitochondria in *Drosophila*. Confocal microscopy observations revealed that Bcl-rambo primarily co-localized with MitoTracker (Fig 1B). In contrast, Bcl-rambo (ΔTM) was uniformly distributed over the cytoplasm (Fig 1B). Consistent with this, Bcl-rambo (1–441) and Bcl-rambo (1–459) did not localize to the mitochondria and exhibited a cytoplasmic distribution (panel B in S1 Fig). The localization of Bcl-rambo and Bcl-rambo (ΔTM) was further analyzed by subcellular fractionation. The mitochondrial chaperone HSP60 was present in the P10 fraction and, to a lesser extent, in the S100 fraction, while α-tubulin was only present in the S100 fractions (Fig 1C), indicating that the P10 fraction was enriched with mitochondria. Similar to HSP60, Bcl-rambo was present mainly in the P10 fraction and marginally in the S100 fraction, whereas Bcl-rambo (ΔTM) was only present in the S100 fraction (Fig 1C). These results indicated that Bcl-rambo mainly localized to mitochondria in *Drosophila* S2 cells.

### Bcl-rambo induced apoptosis in *Drosophila* S2 cells

**Fig 2:**
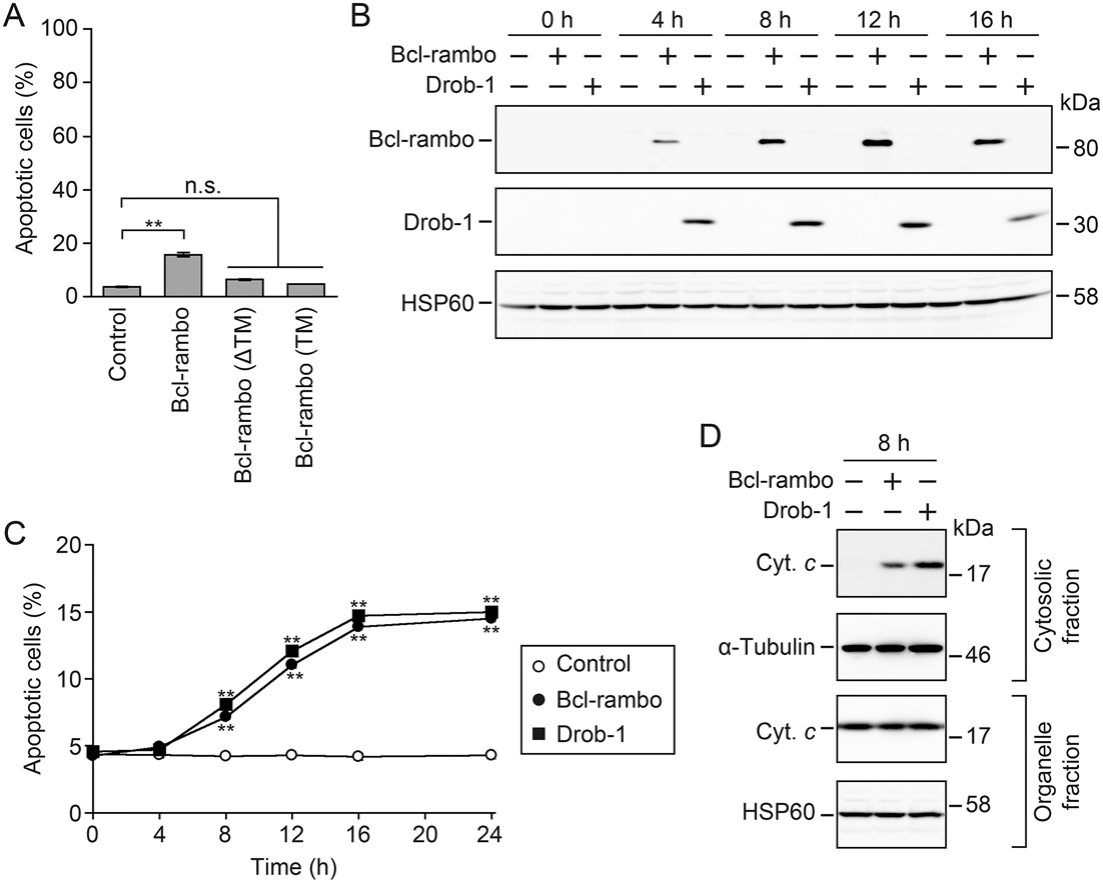
Bcl-rambo induced apoptosis in *Drosophila* S2 cells. (A) S2 cells were transfected with pAct5C-*GAL4* together with pUAST, pUAST-*Bcl-rambo*, pUAST-*Bcl-rambo (ΔTM)*, pUAST-*Bcl-rambo (TM)*, or pUAST-*DsRed-monomer* for 24 h. Cells were stained with Hoechst 33342. Nuclear morphology was observed by fluorescent microscopy. Apoptotic cells (%) are shown as the mean ± S.E. of three independent experiments. **P<0.01, significantly different from the control. n.s., not significant. Transfection efficiency was measured by counting DsRed-monomer-expressing cells, and calculated to be 17.0 ± 0.7% (the mean ± S.E of three independent experiments). (B) S2 cells were transfected with (+) or without (–) pMT-V5-His A/*Bcl-rambo* (no tag), or pMT-V5-His A/*Drob-1* (FLAG tag) for 20 h and then incubated in the presence of CuSO_4_ (500 μM) for the indicated times. The expression of Bcl-rambo and Drob-1 was analyzed by Western blotting using anti-Bcl-rambo and anti-FLAG antibodies, respectively. Data were representative of three independent experiments. (C) S2 cells were transfected with pMT-V5-His A (open circles), pMT-V5-His A/*Bcl-rambo* (filled circles), or pMT-V5-His A/*Drob-1* (filled squares) for 20 h and then incubated in the presence of CuSO_4_ (500 μM) for the indicated times. Cells were stained with Hoechst 33342. Apoptotic cells (%) are shown as the mean ± S.E. of three independent experiments. **P<0.01, significantly different from the control. (D) S2 cells were transfected with (+) or without (–) pMT-V5-His A/*Bcl-rambo* or pMT-V5-His A/*Drob-1* for 20 h and then incubated in the presence of CuSO_4_ (500 μM) for 8 h. The cytosolic fraction and organelle fraction containing mitochondria were both analyzed by Western blotting using anti-cytochrome *c* (Cyt. *c*), α-tubulin, and anti-HSP60 antibodies. Data are representative of two independent experiments.

Previous studies reported that the overexpression of Bcl-rambo induced apoptosis or promoted etoposide- and taxol-induced cell death in mammalian cells [12,19–22]. S2 cells were transiently transfected with pUAST-*Bcl-rambo*, pUAST-*Bcl-rambo (ΔTM)* or pUAST-*Bcl-rambo (TM)* together with pAct5C-*GAL4* to investigate whether Bcl-rambo induced apoptosis in *Drosophila*. We found that Bcl-rambo significantly induced apoptosis, while Bcl-rambo (ΔTM) or Bcl-rambo (TM) only very weakly induced apoptosis (Fig 2A). Since transfection efficiency was calculated to be approximately 17%, it appeared that most S2 cells transfected with Bcl-rambo underwent apoptosis. Moreover, Bcl-rambo (1–441) and Bcl-rambo (1–459) did not induce apoptosis in S2 cells (S2 Fig).

We further investigated the kinetics of apoptosis induced by human Bcl-rambo and the *Drosophila* pro-apoptotic Bcl-2 family member Drob-1/Debcl/dBorg-1/DBok [32–35]. In S2 cells transiently transfected with expression vectors driven by the metallothionein promoter, the expression of Bcl-rambo and Drob-1 was induced within 4 h of the CuSO_4_ treatment, and their expression levels were maintained at least up to 16 h (Fig 2B). The ectopic expression of Bcl-rambo or Drob-1 initiated the induction of apoptosis during 4–8 h, and the number of apoptotic cells steadily increased up to 16 h (Fig 2C). Cytochrome *c* was detected in the cytosolic fraction within 8 h of the CuSO_4_ treatment in Bcl-rambo- or Drob-1-transfected S2 cells, but not control S2 cells (Fig 2D). These results suggest that Bcl-rambo induces the mitochondrial pathway of apoptosis in *Drosophila*.

### Ectopic expression of Bcl-rambo, but not Bcl-rambo (ΔTM), induced morphological aberrations in *Drosophila* tissues

**Fig 3:**
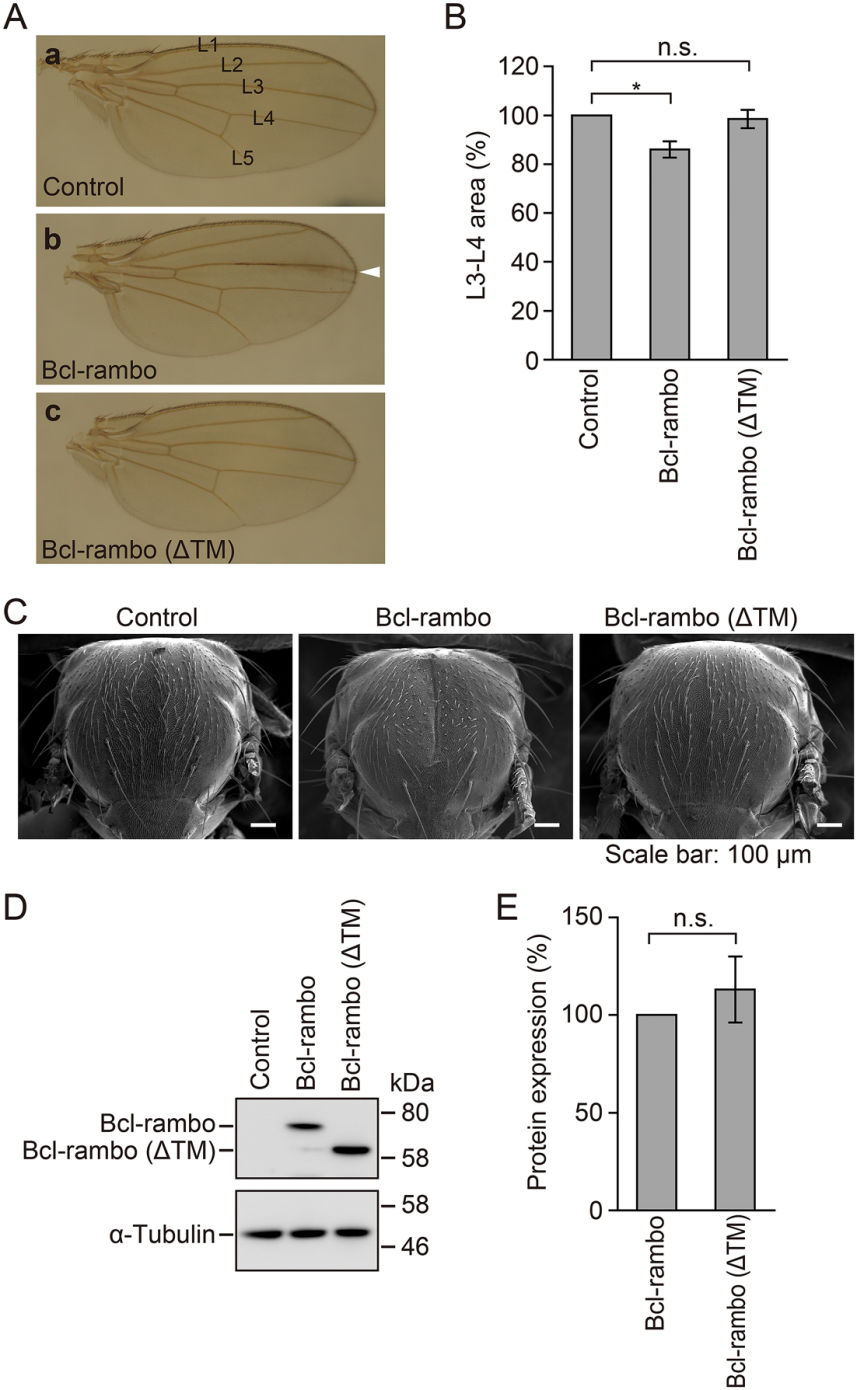
Ectopic expression of Bcl-rambo, but not Bcl-rambo (ΔTM), induced aberrant morphological changes in *Drosophila*. (A and B) Bcl-rambo and Bcl-rambo (ΔTM) were expressed using *dpp*-*GAL4* driver fly lines. (a) *w*; +; *dpp-GAL4*/+, (b) *w*; *UAS-Bcl-rambo*/+; *dpp-GAL4*/+, (c) *w*; *UAS-Bcl-rambo(ΔTM)/+*; *dpp-GAL4*/+. The morphology of the wings was observed under light microscopy. The white arrow indicates morphological aberrations in the wing vein. The L3-L4 area was measured by Image J software (B). Data are shown as the mean ± S.E. of three independent experiments (n = 4–5 for each experiment). **P<0.05, significantly different from control. n.s., not significant. (C) Bcl-rambo and Bcl-rambo (ΔTM) were expressed using *pnr*-*GAL4* driver lines. (a) *w*; +; *pnr-GAL4*/+, (b) *w*; *UAS-Bcl-rambo*/+; *pnr-GAL4*/+, (c) *w*; *UAS-Bcl-rambo (ΔTM)/+*; *pnr-GAL4/+*. The morphology of adult thoraxes was observed by SEM. Scale bars indicate 100 μm. Data were representative of three independent experiments. (D and E) Cell lysates of third instar larval salivary glands were prepared from *sg-GAL4/w*; +; +, *sg-GAL4/w*; *UAS-Bcl-rambo/+*; +, and *sg-GAL4/w*; *UAS-Bcl-rambo (ΔTM)/*+; + flies, and analyzed by Western blotting. The amount of Bcl-rambo and Bcl-rambo (ΔTM) was normalized to that of α-tubulin. The protein expression of Bcl-rambo (%) is shown as the mean ± S.E. of three independent experiments.

In order to gain an insight into the function of Bcl-rambo, it was ectopically overexpressed in several tissues of *Drosophila* by the GAL4-UAS expression system. GAL4 is expressed along the anterior-posterior compartment boundary under the control of the *dpp* (*blk*) promoter in the *dpp-GAL4* driver line [36,37]. The ectopic expression of Bcl-rambo using the *dpp*-*GAL4* driver fly lines induced morphological changes in a wing vein (Fig 3A). In particular, the L3-L4 area of Bcl-rambo flies became smaller than that of control (Fig 3B). In contrast, the overexpression of Bcl-rambo (ΔTM) did not cause obviously different phenotypes from those of control (Fig 3A and 3B). In the *pnr-GAL4* driver line, GAL4 is expressed in the dorsal mesothorax [38,39]. The expression of Bcl-rambo using the *pnr*-*GAL4* driver fly lines induced a split-thorax phenotype, while no significant differences were observed between control and Bcl-rambo (ΔTM) (Fig 3C). The *en* gene is expressed in the posterior compartment of each segment of the embryo and plays an essential role in the development stages of *Drosophila*. The overexpression of Bcl-rambo using the *en*-*GAL4* driver fly lines induced embryonic lethality, and no adult flies expressing Bcl-rambo were observed (Table 1). In contrast, Bcl-rambo (ΔTM) did not affect the number of adult flies (Table 1). In *Drosophila*, salivary gland is suitable for preparing cell lysates without the contamination of other tissues. The amount of Bcl-rambo and Bcl-rambo (ΔTM) at the protein level was almost equivalent in the *sg*-*GAL4* driver fly lines (Fig 3D and 3E). Thus, these results indicated that the ectopic expression of Bcl-rambo interfered with the proper differentiation of the wing and thorax as well as embryonic development.

**Table 1:** Phenotypes of transgenic flies expressing *Bcl-rambo* or *Bcl-rambo (ΔTM)*

GAL4 driver	Name of Transgene	Strain	Chromosome	Phenotype
*dpp*	*Bcl-rambo*	#77	II	Atrophied wing
*dpp*	*Bcl-rambo (ΔTM)*	#45	II	n.d.
*pnr*	*Bcl-rambo*	#77	II	Split thorax
*pnr*	*Bcl-rambo (ΔTM)*	#45	II	n.d.
*GMR*	*Bcl-rambo*	#1	III	Rough eye
*GMR*	*Bcl-rambo*	#6	X	Rough eye
*GMR*	*Bcl-rambo*	#46	II	Rough eye
*GMR*	*Bcl-rambo*	#77	II	Rough eye
*GMR*	*Bcl-rambo (ΔTM)*	#10	II	n.d.
*GMR*	*Bcl-rambo (ΔTM)*	#26	II	n.d.
*GMR*	*Bcl-rambo (ΔTM)*	#45	II	n.d.
*GMR*	*Bcl-rambo (ΔTM)*	#148	X	n.d.
*en*	*Bcl-rambo*	#4	X	Lethal
*en*	*Bcl-rambo*	#7	III	Lethal
*en*	*Bcl-rambo*	#46	II	Lethal
*en*	*Bcl-rambo*	#77	II	Lethal
*en*	*Bcl-rambo (ΔTM)*	#22	X	n.d.
*en*	*Bcl-rambo (ΔTM)*	#45	II	n.d.
*en*	*Bcl-rambo (ΔTM)*	#112	III	n.d.

n.d.: no detectable phenotype

### Ectopic expression of Bcl-rambo, but not Bcl-rambo (ΔTM), induced a rough eye phenotype in *Drosophila*

**Fig 4:**
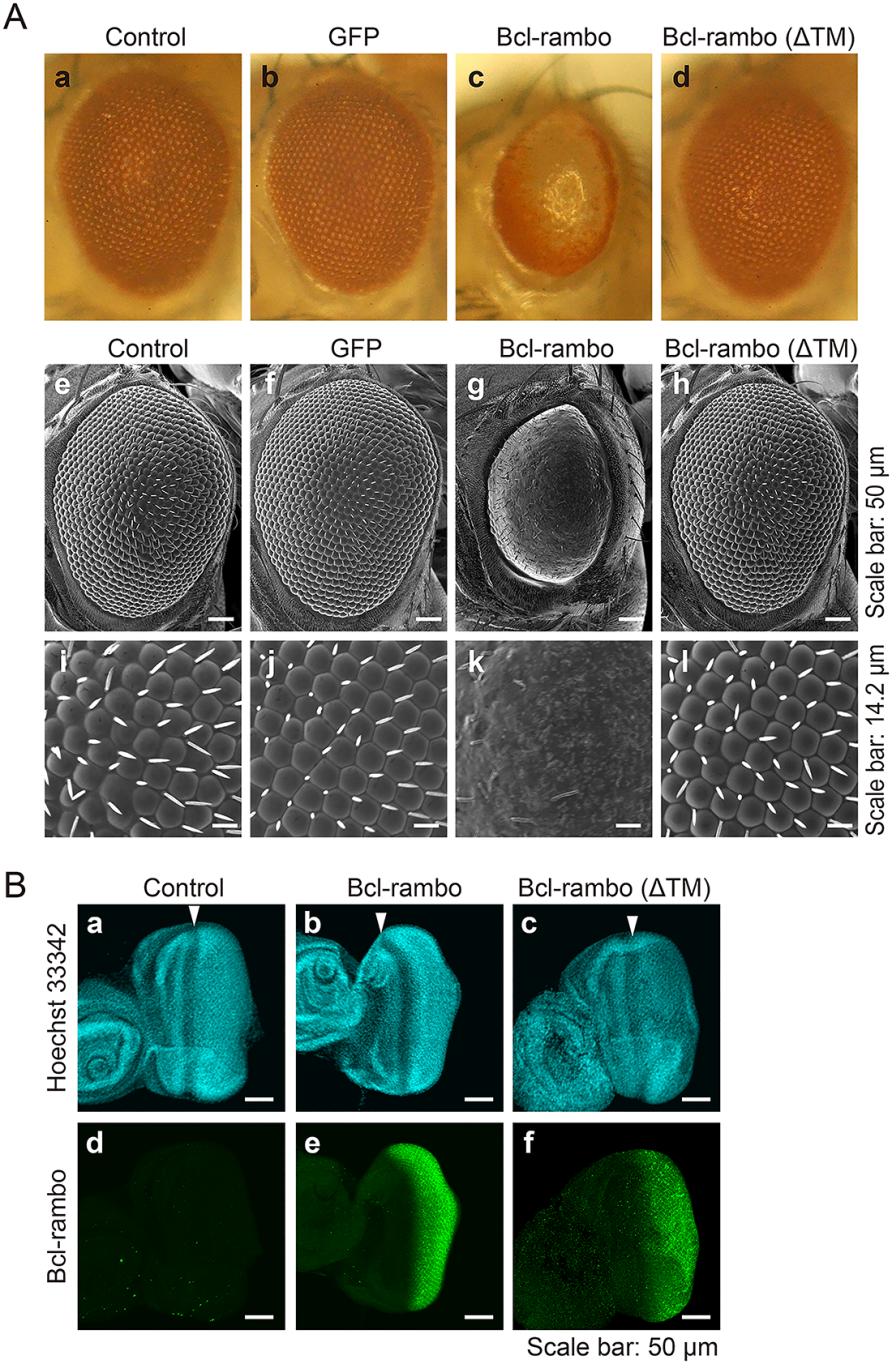
Ectopic expression of Bcl-rambo, but not Bcl-rambo (ΔTM), induced a rough eye phenotype in *Drosophila*. (A) Bcl-rambo and Bcl-rambo (ΔTM) were expressed using *GMR*-*GAL4* driver fly lines. (a, e, i) *GMR-GAL4*/*w*; +/*CyO*; +, (b, f, j) *GMR-GAL4*/*w*; *UAS-GFP*/+; +, (c, g, k) *GMR-GAL4*/*w*; *UAS-Bcl-rambo*/*CyO*; +, (d, h, l) *GMR-GAL4*/*w*; *UAS-Bcl-rambo (ΔTM)*/*CyO*; +. The morphology of adult eyes was observed by light microscopy (a–d) and SEM (e–l). Scale bars in e–h and i–l indicate 50 μm and 14.2 μm, respectively. Light microscopy and SEM photographs were taken from different individuals. Data were representative of three independent experiments. (B) Eye imaginal discs were stained for Bcl-rambo (green) and with Hoechst 33342 (blue). (a and d) *GMR-GAL4*/*w*; +/*CyO* or *Sp*; +, (b and e) *GMR-GAL4*/*w*; *UAS-Bcl-rambo*/*CyO* or *Sp*; +, (c and f) *GMR-GAL4/w*; *UAS-Bcl-rambo (ΔTM)*/*CyO* or *Sp*; +. White arrows show the morphogenetic furrow. Scale bars indicate 50 μm. Data were representative of three independent experiments.

The expression of Bcl-rambo using the *GMR*-*GAL4* driver fly lines induced a rough eye phenotype, such as a reduction in eye size and the loss of ommatidia, bristles, and pigmentation (Fig 4A). In contrast, Bcl-rambo (ΔTM) did not exert any phenotypic changes from control and green fluorescent protein (GFP) (Fig 4A). Bcl-rambo and Bcl-rambo (ΔTM) were similarly expressed in eye imaginal discs (Fig 4B), excluding the possibility that Bcl-rambo (ΔTM) was not or insufficiently expressed. A rough eye phenotype was also observed in other independent transgenic lines for Bcl-rambo, but not Bcl-rambo (ΔTM) (Table 1 and S3 Fig), which ruled out position effects.

### Ectopic expression of Bcl-rambo induced activation of effector caspases in *Drosophila*

**Fig 5:**
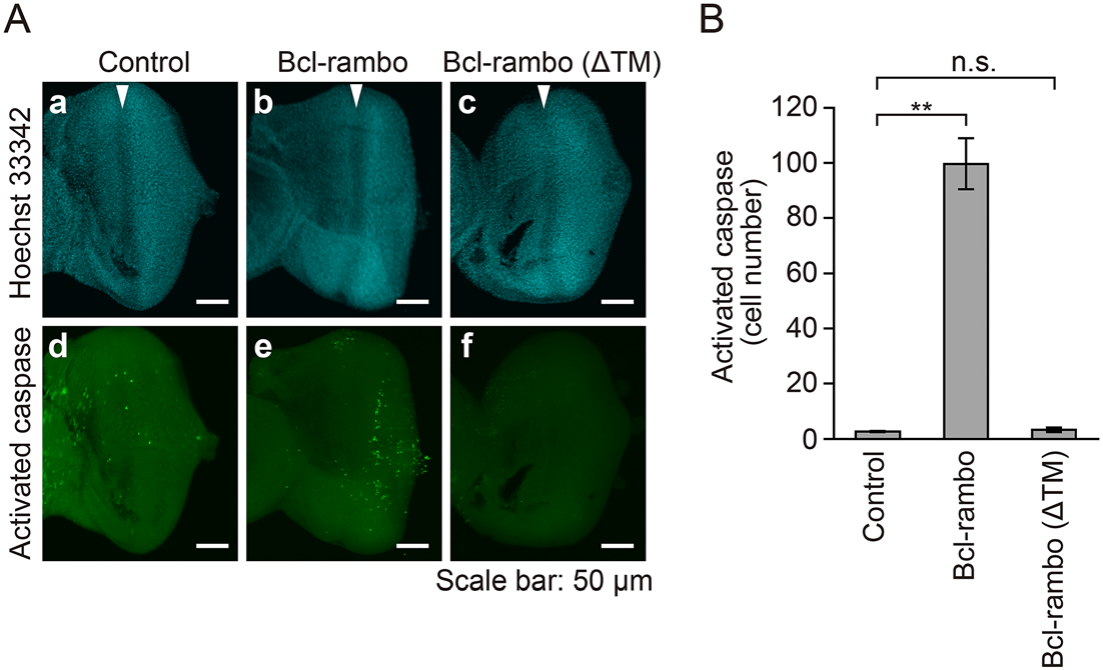
Ectopic expression of Bcl-rambo, but not Bcl-rambo (ΔTM), induced activation of effector caspases in eye imaginal discs. (A and B) (a, d) *GMR-GAL4*/*w*; +/*CyO* or *Sp*; +, (b, e) *GMR-GAL4*/*w*; *UAS-Bcl-rambo*/*CyO* or *Sp*; +, (c, f) *GMR-GAL4*/*w*; *UAS-Bcl-rambo (ΔTM)*/*CyO* or *Sp*; +. The eye imaginal discs were labeled for activated caspase-3/7 (green) and with Hoechst 33342 (blue). Scale bars indicate 50 μm. White arrows indicate the morphogenetic furrow. The number of fluorescent cells harboring activated caspases from the morphogenetic furrow to the posterior region of imaginal eye discs was measured (B). Data are shown as the mean ± S.E. of three independent experiments (n = 6 for each experiment). **P<0.01, significantly different from control. n.s., not significant.

Bcl-rambo was found to induce apoptosis in S2 cells (Fig 2). *Drosophila* effector caspases have been shown to exhibit DEVD-cleaving activity in a manner similar to mammalian effector caspases [40,41]. In order to determine whether the ectopic expression of Bcl-rambo caused caspase activation, a fluorogenic substrate for caspase-3/7 was used to visualize activated effector caspases. When Bcl-rambo was expressed in eye imaginal discs, the activated effector caspases greatly increased from the posterior region to the morphogenetic furrow (Fig 5A and 5B). However, no obvious signals of activated caspases were detected when Bcl-rambo (ΔTM) was expressed (Fig 5A and 5B). These results indicated that the ectopic expression of Bcl-rambo, but not Bcl-rambo (ΔTM), significantly induced the activation of effector caspases.

### Reduced pigmentation induced by Bcl-rambo was rescued by p35, Diap-1, and Diap-2

**Fig 6:**
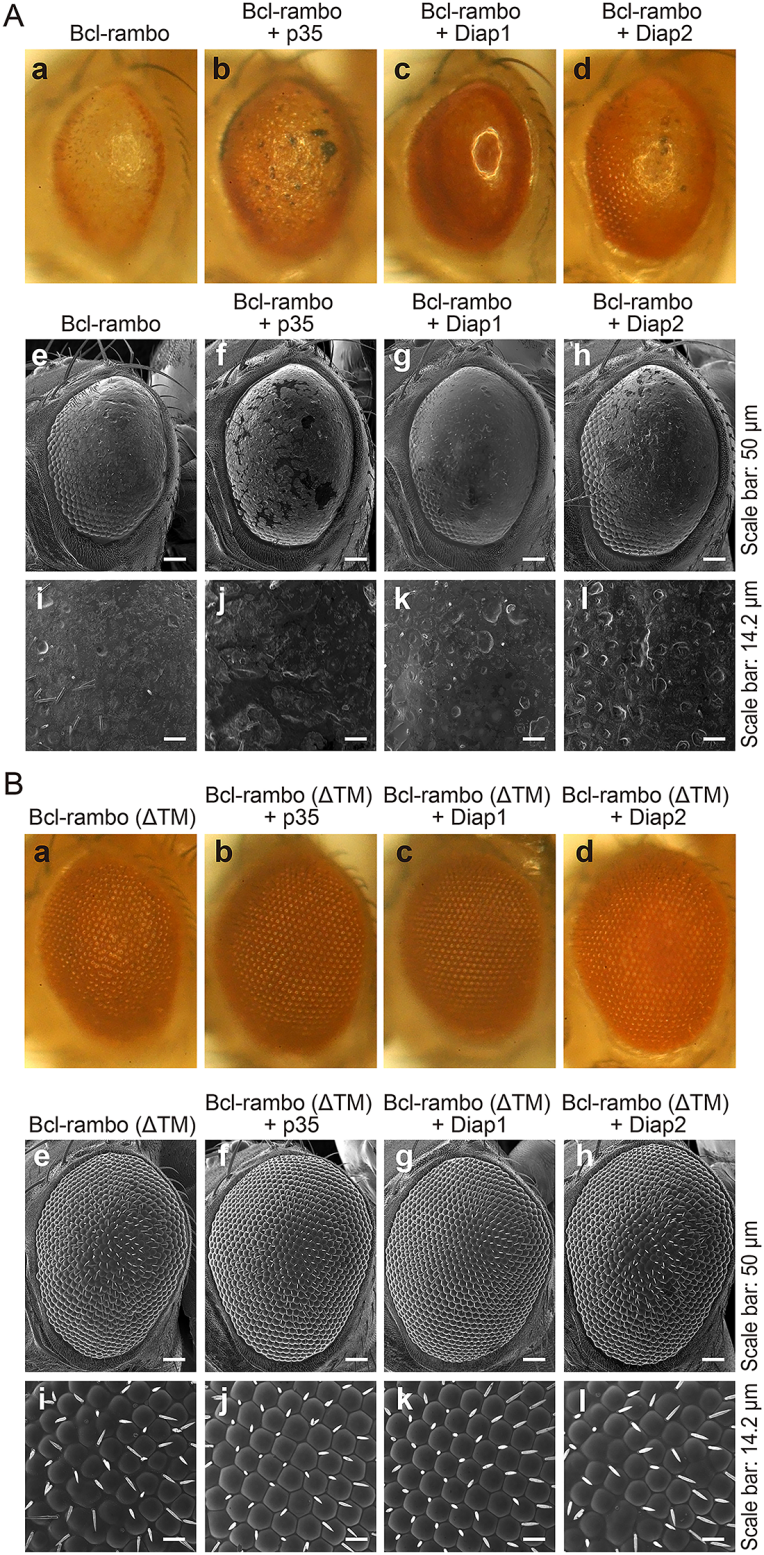
Reduced pigmentation induced by Bcl-rambo was rescued by co-expression of p35, Diap1, and Diap2. (A and B) Bcl-rambo and Bcl-rambo (ΔTM) were expressed using *GMR*-*GAL4* driver fly lines. (A) (a, e, i) *GMR-GAL4*/*w*; *UAS-Bcl-rambo*/+; +, (b, f, j) *GMR-GAL4*/*w*; *UAS-Bcl-rambo*/+; *UAS-p35*/+, (c, g, k) *GMR-GAL4*/*w*; *UAS-Bcl-rambo*/+; *UAS-Diap1*/+, (d, h, l) *GMR-GAL4/w*; *UAS-Bcl-rambo/+*; *GMR-Diap2/+*. (B) (a, e, i) *GMR-GAL4/w*; *UAS-Bcl-rambo (ΔTM)/+*; +, (b, f, j) *GMR-GAL4/w*; *UAS-Bcl-rambo (ΔTM)/+*; *UAS-p35/+*, (c, g, k) *GMR-GAL4/w*; *UAS-Bcl-rambo (ΔTM)/+*; *UAS-Diap1/+*, (d, h, l) *GMR-GAL4/w*; *UAS-Bcl-rambo (ΔTM)/+*; *GMR-Diap2/+*. The morphology of adult eyes was observed by light microscopy (a–d) and SEM (e–l). Scale bars in e–h and i–l indicate 50 μm and 14.2 μm, respectively. Light microscopy and SEM photographs were taken from different individuals. Data were representative of three independent experiments.

The baculovirus p35 and *Drosophila* Diap1 and Diap2 have been shown to inhibit caspase activity and regulate caspase-dependent apoptosis [28,42]. We further investigated whether the rough eye phenotype induced by Bcl-rambo was rescued by p35, Diap1, and Diap2. The reduction in eye pigments induced by Bcl-rambo was rescued by the co-expression of p35, Diap1, and Diap2 (Fig 6A). In contrast, the co-expression of p35, Diap1, and Diap2 only weakly suppressed the abnormal formation of ommatidia and bristles as well as the reduction in eye size induced by Bcl-rambo (Fig 6A). Bcl-rambo (ΔTM) did not cause any changes with the co-expression of p35, Diap1, and Diap2 (Fig 6B). Moreover, additional UAS repeats in the UAS*-GFP* insertion did not rescue the rough eye phenotype induced by Bcl-rambo (S4 Fig), excluding the effect of diluting *GMR-GAL4*.

### Aberrant morphology of ommatidia induced by Bcl-rambo was partly rescued by p35

**Fig 7:**
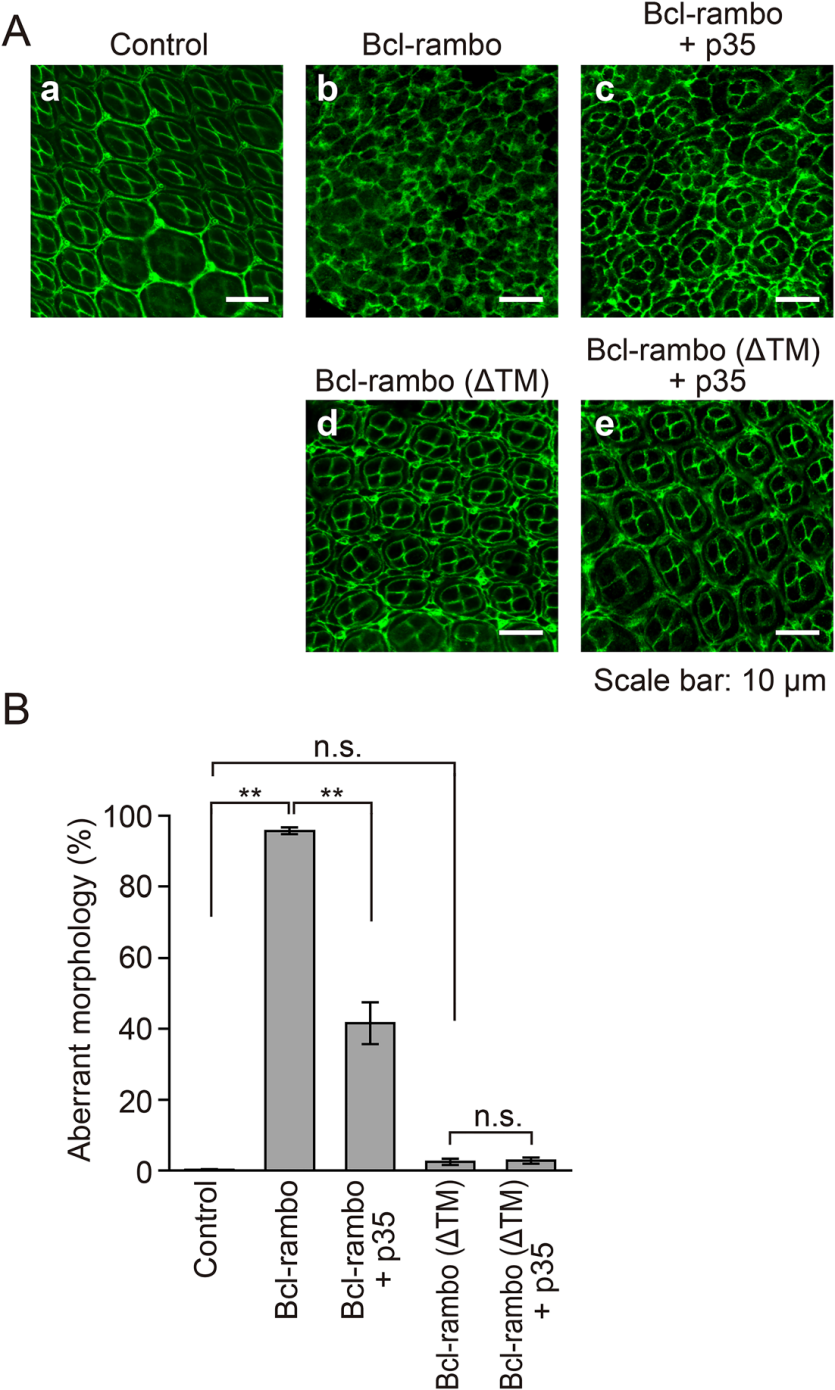
Aberrant morphological changes in ommatidia induced by Bcl-rambo were partly rescued by p35. (A and B) Pupal retinae 42 h after pupal formation were stained with an anti-discs large antibody (green). (a) *w/+*; +; +, (b) *GMR-GAL4*/*w*; *UAS-Bcl-rambo*/+; +, (c) *GMR-GAL4*/*w*; *UAS-Bcl-rambo*/+; *UAS-p35*/+, (d) *GMR-GAL4*/*w*; *UAS-Bcl-rambo (ΔTM)*/+; +, (e) *GMR-GAL4*/*w*; *UAS-Bcl-rambo (ΔTM)*/+; *UAS-p35*/+. Scale bars indicate 10 μm. The numbers of normal and aberrant ommatidia were counted. Aberrant morphology (%) is shown as the mean ± S.E. of three independent experiments (n = 5 for each experiment). **P<0.01. n.s., not significant.

The *Drosophila* compound eye is composed of eight photoreceptor cells, four cone cells, two primary pigment cells, six secondary pigment cells, three tertiary pigment cells, and three mechanosensory bristles. The differentiation of these cells was completed ~42 h after pupal formation at 28°C. Since the loss-of-pigment phenotype induced by Bcl-rambo was effectively rescued by the co-expression of caspase inhibitors, we examined the morphology of the *Drosophila* pupal retina 42 h after pupal formation. When Bcl-rambo was expressed using the *GMR-GAL4* driver fly lines, the shapes of the cone cells and pigment cells were markedly altered because four cone cells per ommatidium and the surrounding pigment cells were hardly distinguishable (Fig 7A and 7B). In contrast, the morphology of the pupal retina expressing Bcl-rambo (ΔTM) was not obviously changed, but showed a slight defect in the ommatidia rotation (Fig 7A). The aberrant morphology of ommatidia induced by Bcl-rambo was partly rescued by p35 (Fig 7A and 7B).

We further investigated whether Bcl-rambo induced apoptosis in the pupal retinae at the same period. The signals for activated caspase-3/7 were rarely detected in the control pupal retinae, and they were not increased in the pupal retinae when Bcl-rambo or Bcl-rambo (ΔTM) was expressed (data not shown). These results suggest that Bcl-rambo does not induce apoptosis in the pupal retinae at this period.

**Fig 8:**
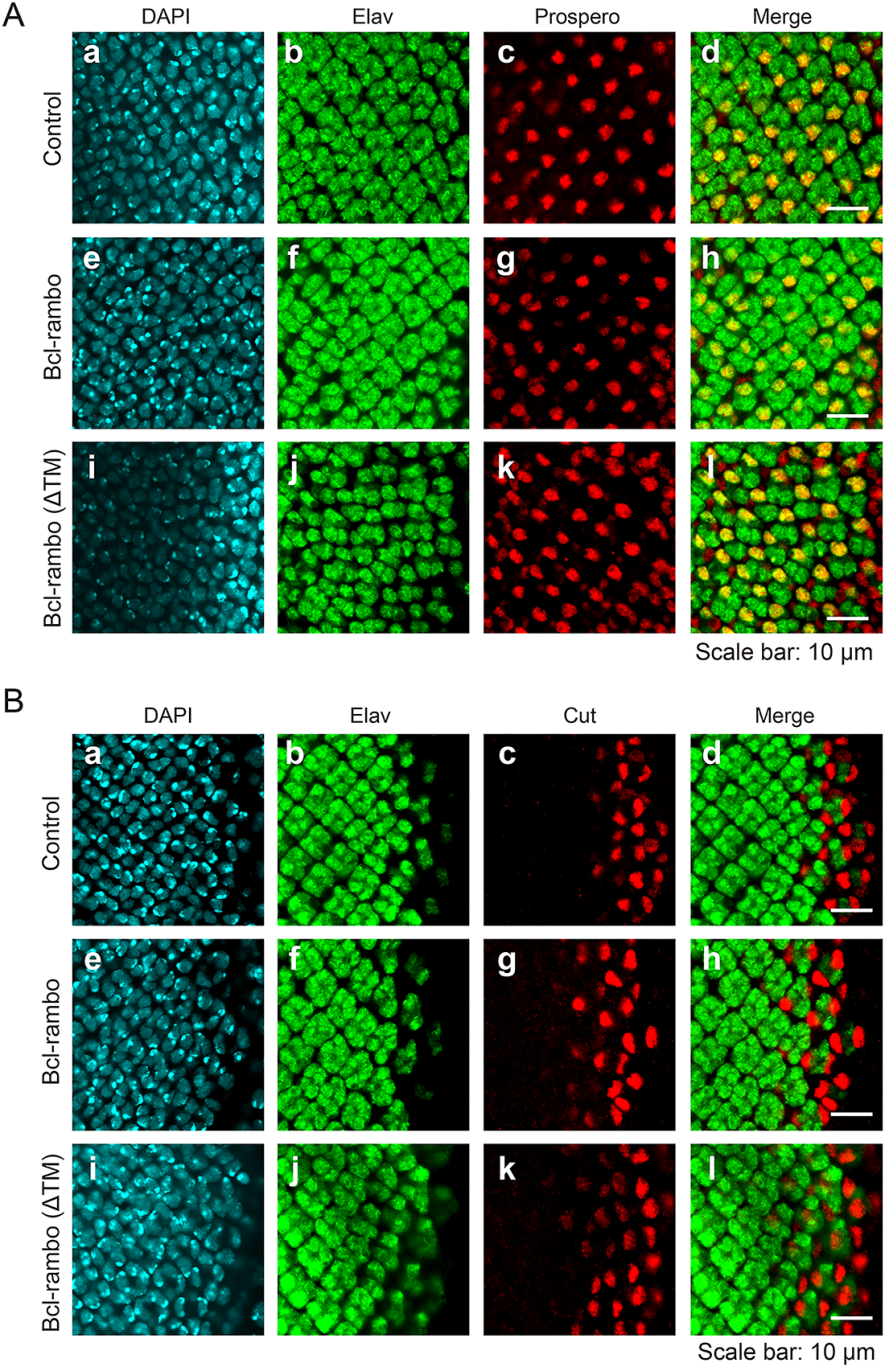
Bcl-rambo did not affect the expression of Cut, Elav, or Prospero. (A and B) Eye imaginal discs were stained for DAPI (blue) and Elav (green), together with Prospero (red) (A) or Cut (red) (B). (a–d) *GMR-GAL4*/*w*; +/*CyO* or *Sp*; +, (e–h) *GMR-GAL4*/*w*; *UAS-Bcl-rambo*/*CyO* or *Sp*; +, (i–l) *GMR-GAL4/w*; *UAS-Bcl-rambo (ΔTM)*/*CyO* or *Sp*; +. Scale bars indicate 10 μm. Data were representative of at least two independent experiments.

In order to determine whether Bcl-rambo affected eye development, eye imaginal discs were stained with Elav for a marker of all photoreceptor cells, Prospero for a marker of R7 cells, and Cut for a maker of cone cell precursors. Elav, Prospero, and Cut were expressed in eye imaginal discs even when Bcl-rambo or Bcl-rambo (ΔTM) was expressed (Fig 8A and 8B). These results suggested that Bcl-rambo did not affect the differentiation of photoreceptor cells in the eye discs of third instar larvae. Although the formation of Cut-positive cone cell precursors was not affected by Bcl-rambo (Fig 8B), the differentiation of cone cells and pigment cells were clearly affected by the expression of Bcl-rambo in pupal retinae (Fig 7A). The spacing of Elav clusters appeared to be abnormal (Fig 8A and 8B), and this may have been due to the death of interommatidial cells. Therefore, the primary effect of Bcl-rambo appears to be the induction of apoptosis, while defects in the differentiation of cone cells and pigment cells may be secondary effects during the later stage of eye development.

### Bcl-rambo did not interact with Drob-1 or Buffy

**Fig 9:**
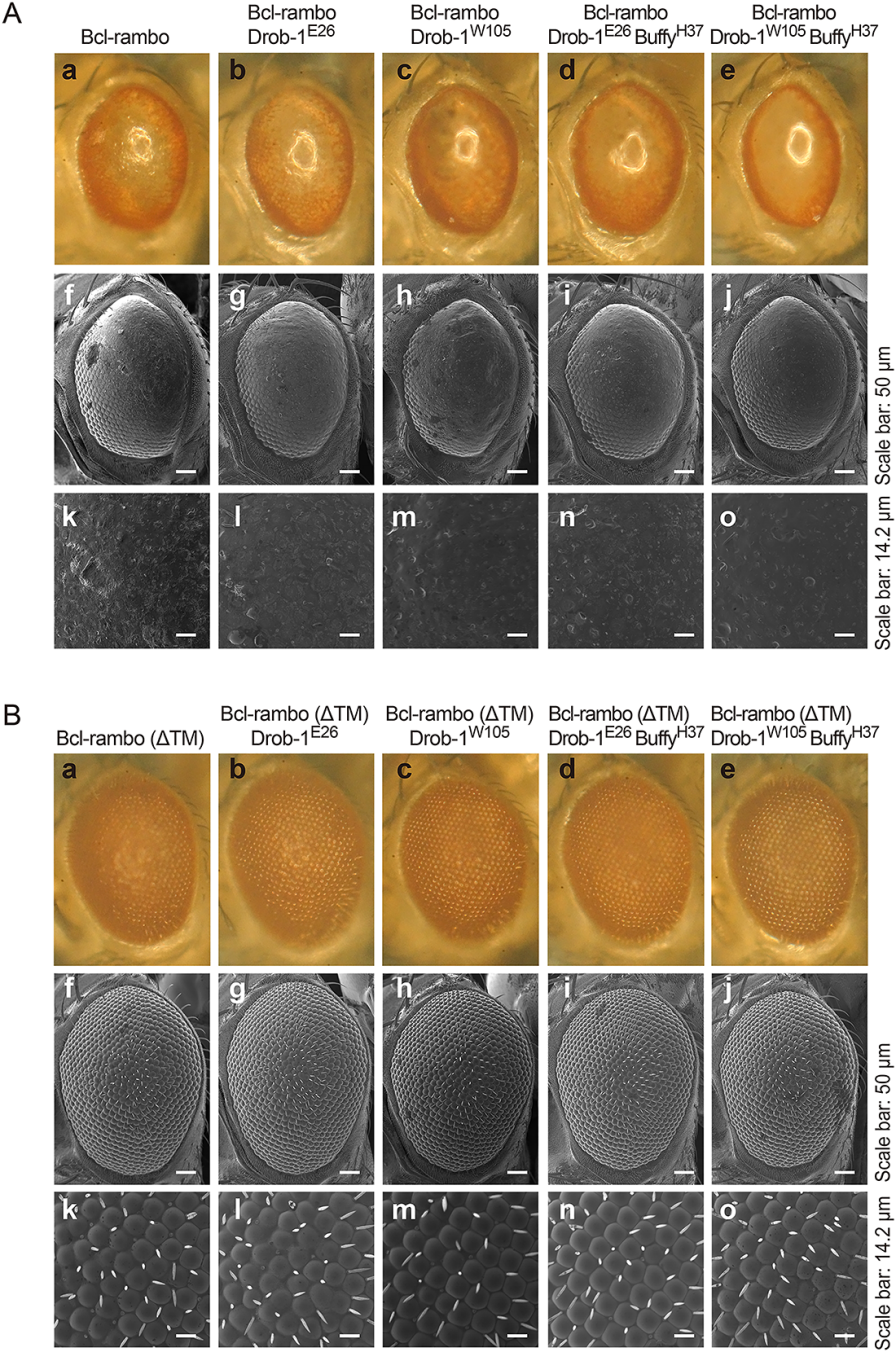
Bcl-rambo did not interact genetically with Drob-1 or Buffy. (A and B) Bcl-rambo and Bcl-rambo (ΔTM) were expressed using *GMR*-*GAL4* driver fly lines. (A) (a, f, k) *GMR-GAL4*/*w*; *UAS-Bcl-rambo*/+; +, (b, g, l) *GMR-GAL4*/*w*; *UAS-Bcl-rambo*/*Drob-1*^*E26*^; +, (c, h, m) *GMR-GAL4*/*w*; *UAS-Bcl-rambo*/*Drob-1*^*W105*^; +, (d, i, n) *GMR-GAL4/w*; *UAS-Bcl-rambo/Drob-1*^*E26*^ *Buffy*^*H37*^; +, (e, j, o) *GMR-GAL4/w*; *UAS-Bcl-rambo/Drob-1*^*W105*^ *Buffy*^*H37*^; +. (B) (a, f, k) *GMR-GAL4/w*; *UAS-Bcl-rambo (ΔTM)/+*; +, (b, g, l) *GMR-GAL4/w*; *UAS-Bcl-rambo (ΔTM)/Drob-1*^*E26*^; +, (c, h, m) *GMR-GAL4/w*; *UAS-Bcl-rambo (ΔTM)/Drob-1*^*W105*^; +, (d, i, n) *GMR-GAL4/w*; *UAS-Bcl-rambo (ΔTM)/Drob-1*^*E26*^ *Buffy*^*H37*^; +, (e, j, o) *GMR-GAL4/w*; *UAS-Bcl-rambo (ΔTM)/Drob-1*^*W105*^ *Buffy*^*H37*^; +. The morphology of the adult eyes was observed by light microscopy (a–e) and SEM (f–o). Scale bars in f–j and k–o indicate 50 μm and 14.2 μm, respectively. Data were representative of three independent experiments.

*Drosophila* has two Bcl-2 family proteins: Drob-1/Debcl and Buffy/dBorg-2, both of which have been classified into pro-apoptotic multidomain proteins, and are closely related to mammalian Bok [32–35]. We investigated whether Bcl-rambo interacted genetically with Drob-1 and Buffy. The rough eye phenotype induced by Bcl-rambo was little if any influenced by the *Drob-1*^*E26*^ or *Drob-1*^*W105*^ mutation or by the *Drob-1*^*E26*^ *Buffy*^*H37*^ or *Drob-1*^*W105*^ *Buffy*^*H37*^ mutation (Fig 9A). Bcl-rambo (ΔTM) did not exhibit any phenotypic changes in *Drob-1*^*E26*^ *Buffy*^*H37*^, or *Drob-1*^*W105*^ *Buffy*^*H37*^ flies (Fig 9B). Moreover, the *Buffy*^*H37*^ mutation did not influence the rough eye phenotype induced by Bcl-rambo (S5 Fig).

**Fig 10:**
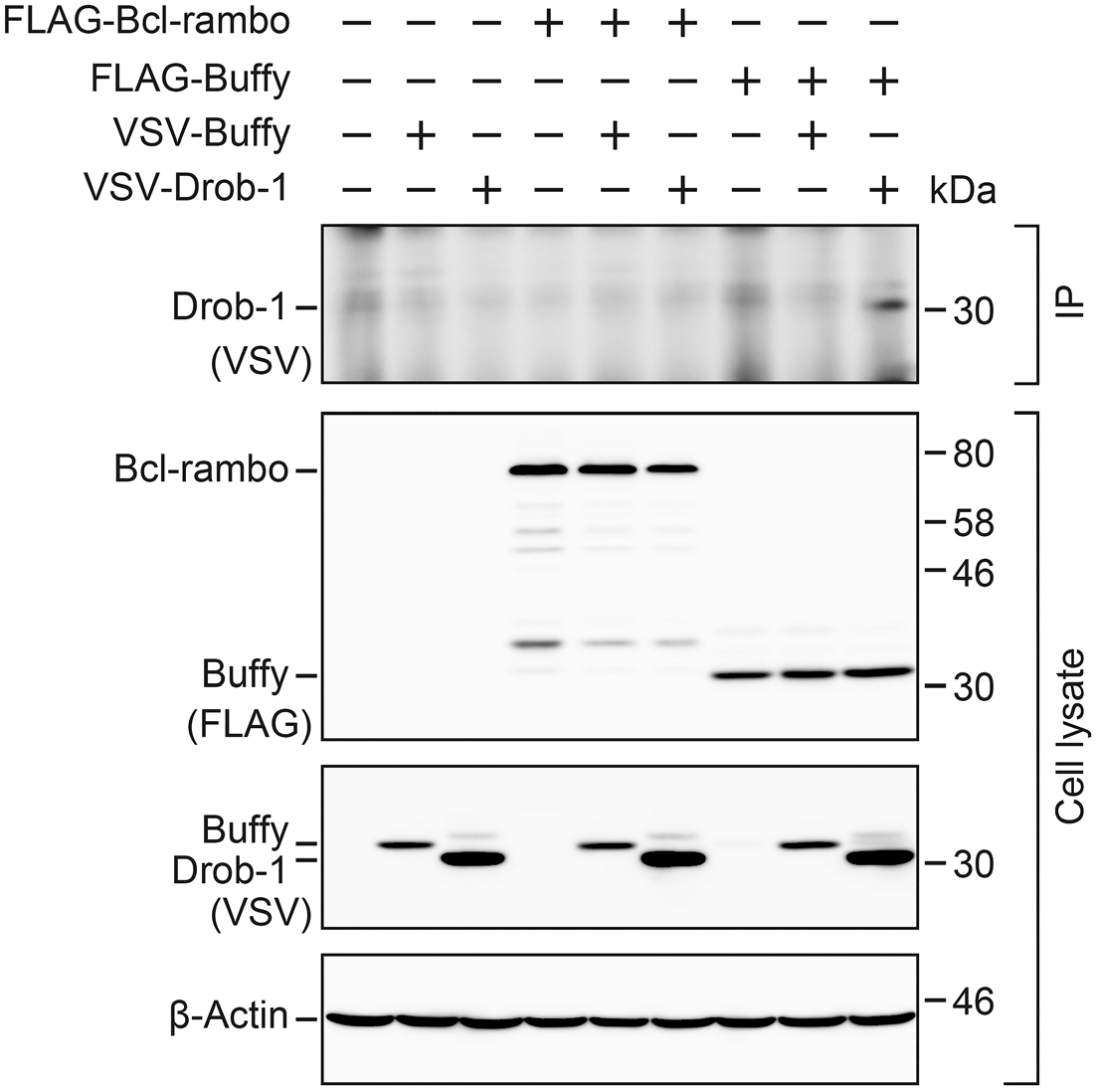
Bcl-rambo did not bind to Drob-1/Debcl or Buffy. HEK293T cells were transfected with (+) or without (–) expression vectors for FLAG-tagged Bcl-rambo or FLAG-tagged Buffy together with VSV-tagged Buffy or VSV-tagged Drob-1/Debcl for in the presence of zVAD-fmk (20 μM) for 16 h. Cell lysates were immunoprecipitated with anti-FLAG antibody-conjugated beads. Immunoprecipitates (IP) were analyzed by Western blotting using an anti-VSV antibody. Cell lysates were analyzed by Western blotting using anti-FLAG, anti-VSV, and β-actin antibodies. Data were representative of three independent experiments.

An immunoprecipitation assay was performed to confirm the weak genetic interaction between Bcl-rambo and either Drob-1/Debcl or Buffy. FLAG-tagged Bcl-rambo and FLAG-tagged Buffy were transiently overexpressed together with VSV-tagged Drob-1/Debcl or VSV-tagged Buffy in HEK293T cells, and FLAG-tagged proteins were pulled down. Bcl-rambo did not bind to Drob-1/Debcl or Buffy in HEK293T cells, while Buffy bound to Drob-1/Debcl (Fig 10).

### Bcl-rambo interacted genetically with adenine nucleotide translocators and the autophagy-related 8a protein

**Fig 11:**
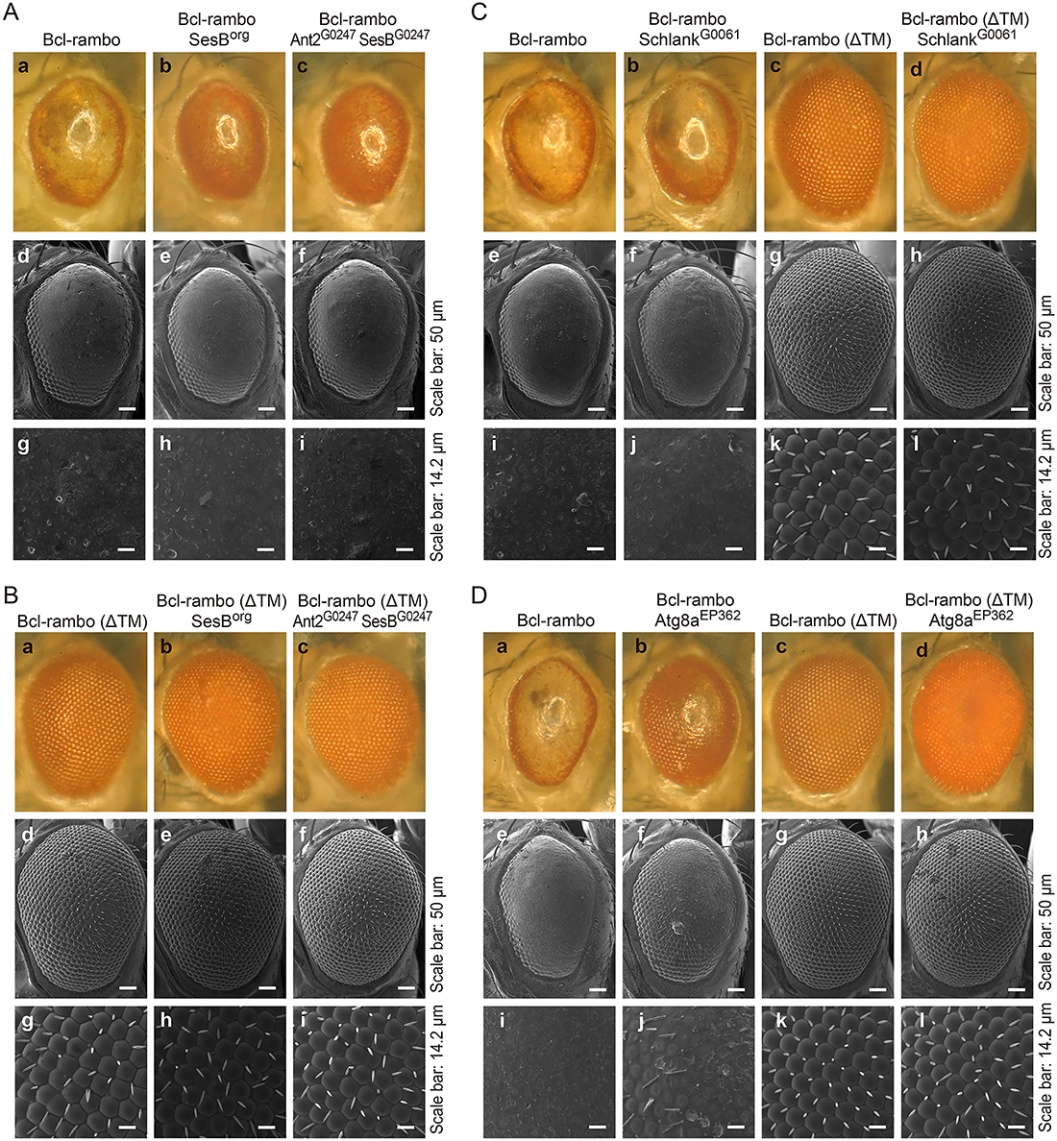
Reduced pigmentation induced by Bcl-rambo was rescued by mutations in adenine nucleotide translocators and Atg8a. (A to C) (A) (a, d, g) *GMR-GAL4*/*w*; *UAS-Bcl-rambo*/+; +, (b, e, h) *sesB*^*org*^/*GMR-GAL4*; *UAS-Bcl-rambo*/+; +, (c, f, i) *w*^*67c23*^ *P{lacW}Ant2*^*G0247*^ *sesB*^*G0247*^/*GMR-GAL4*; *UAS-Bcl-rambo*/+; +. (B) (a, d, g) *GMR-GAL4*/*w*; *UAS-Bcl-rambo (ΔTM)*/+; +, (b, e, h) *sesB*^*org*^/*GMR-GAL4*; *UAS-Bcl-rambo (ΔTM)*/+; +, (c, f, i) *w*^*67c23*^ *P{lacW}Ant2*^*G0247*^ *sesB*^*G0247*^/*GMR-GAL4*; *UAS-Bcl-rambo (ΔTM)*/+; +. The morphology of adult eyes was observed by light microscopy (a–c) and SEM (d–i). (C) (a, e, i) *GMR-GAL4*/*w*; *UAS-Bcl-rambo*/+; +, (b, f, j) *w*^*67c23*^ *P{lacW}schlank*^*G0061*^ *schlank*^*G0061*^/*GMR-GAL4*; *UAS-Bcl-rambo*/+; + (c, g, k) *GMR-GAL4*/*w*; *UAS-Bcl-rambo (ΔTM)*/+; +, (d, h, l) *w*^*67c23*^ *P{lacW}schlank*^*G0061*^ *schlank*^*G0061*^/*GMR-GAL4*; *UAS-Bcl-rambo (ΔTM)*/+; +. The morphology of adult eyes was observed by light microscopy (a–d) and SEM (e–l). Scale bars in e–h and i–l indicate 50 μm and 14.2 μm, respectively. Data were representative of two independent experiments. (D) (a, e, i) *GMR-GAL4*/*w*; *UAS-Bcl-rambo*/+; +, (b, f, j) *w*^*1118*^ *P{EP}Atg8a*^*EP362*^/*GMR-GAL4*; *UAS-Bcl-rambo*/+; + (c, g, k) *GMR-GAL4*/*w*; *UAS-Bcl-rambo (ΔTM)*/+; +, (d, h, l) *w*^*1118*^ *P{EP}Atg8a*^*EP362*^/*GMR-GAL4*; *UAS-Bcl-rambo (ΔTM)*/+; +. The morphology of adult eyes was observed by light microscopy (a–d) and SEM (e–l). Scale bars in e–h and i–l indicate 50 μm and 14.2 μm, respectively. Data were representative of three independent experiments.

Recent studies reported that Bcl-rambo bound to adenine nucleotide translocators [21], ceramide synthases 2 (CerS2) and 6 (CerS6) [17], and LC3, a mammalian homologue of the autophagy-related 8 (Atg8) protein [23]. We herein investigated whether Bcl-rambo interacted genetically with stress-sensitive B (SesB) and Ant2 (a major *Drosophila* homologue of adenine nucleotide translocators and the second isogene, respectively), Schlank (a *Drosophila* homologue of ceramide synthases), and *Drosophila* Atg8a. The loss-of-pigment phenotype induced by Bcl-rambo was effectively rescued by the *sesB*^*org*^ or *Ant2*^*G0247*^ *sesB*^*G0247*^ mutation (Fig 11A), while Bcl-rambo (ΔTM) did not exhibit any phenotypic changes in *sesB*^*org*^ or *Ant2*^*G0247*^ *sesB*^*G0247*^ flies (Fig 11B). The *schlank*^*G0061*^ mutation did not rescue the loss-of-pigment phenotype induced by Bcl-rambo (Fig 11C). The *Atg8a*^*EP362*^ mutation rescued the loss-of-pigmentation induced by Bcl-rambo, and partly suppressed the loss of bristles induced by Bcl-rambo, while Bcl-rambo (ΔTM) flies did not exhibit the clear effects observed in *Atg8a*^*EP362*^ flies (Fig 11D). Due to the insertion of UAS repeats in *Atg8a*^*EP362*^ flies, Atg8a may be overexpressed in the presence of the *GMR-GAL4* driver.

## Discussion

We previously identified human Bcl-rambo and showed that its overexpression induced apoptosis in HEK293T cells [12]. Consistent with these findings, previous studies reported that Bcl-rambo induced apoptosis in other cell lines [20–22]. However, Bcl-rambo has been also shown to inhibit pro-apoptotic ceramide synthases in glioblastoma [17]. Bcl-rambo is known to be strongly expressed in several cancer cells [13–17], implying that Bcl-rambo may inhibit apoptosis or promote growth. Thus, it currently remains unclear whether Bcl-rambo plays either a pro-apoptotic role or anti-apoptotic role or even both roles under physiological conditions. In order to gain an insight into this issue, we investigated the biological activity of human Bcl-rambo in *Drosophila*. The ectopic expression of Bcl-rambo induced apoptosis in *Drosophila* S2 cells. Bcl-rambo also induced apoptosis and morphological aberrations representative of the rough eye phenotype in transgenic flies.

Bcl-rambo, but not Bcl-rambo (ΔTM), mainly localized to mitochondria in *Drosophila* S2 cells. This result was consistent with our previous findings obtained using HEK293T cells [12], and indicated that the C-terminal TM was essential for the mitochondrial localization of Bcl-rambo. A previous study showed that the mitochondrial targeting of Bcl-x_L_ required the C-terminal TM domain to be flanked at both ends by at least two basic amino acids [43]. This consensus sequence proposed for mitochondrial localization was found to be conserved in human Bcl-rambo [12]. *Drosophila* has two Bcl-2 family proteins Drob-1/Debcl and Buffy [32–35]. Drob-1/Debcl has been shown to predominantly localize to mitochondria, whereas Buffy localizes to the endoplasmic reticulum in *Drosophila* and mammalian cell lines [44]. Drob-1/Debcl, but not Buffy, has C-terminal positively-charged residues homologous with Bcl-x_L_ [44]. Thus, Bcl-rambo localizes to mitochondria in a manner similar to Drob-1/Debcl in *Drosophila*.

In *Drosophila*, Bcl-2 family members were found to not play a central role in the regulation of apoptosis [9,26]. Drob-1/Debcl and Buffy are not required for normal development, but participate in stress-induced apoptosis [45]. The ectopic expression of Drob-1/Debcl induced apoptosis in cultured cells and transgenic flies [32–35], indicating that Drob-1/Debcl exhibits pro-apoptotic activity. In contrast, Buffy has an anti-apoptotic function and has been shown to interact genetically and physically with Drob-1/Debcl to suppress Drob-1/Debcl-induced cell death [46]. A previous study reported that Drob-1/Debcl protected neurons from polyglutamine-induced toxicity, while Buffy enhanced it [47]. These findings suggested that Drob-1/Debcl and Buffy possess both pro-apoptotic and anti-apoptotic activities. The ectopic expression of Bcl-rambo, but not Bcl-rambo (ΔTM), induced apoptosis in *Drosophila* S2 cells and caspase activation in eye imaginal discs. This is consistent with our previous findings in which Bcl-rambo induced caspase activation in HEK293T cells [12]. In S2 cells, Bcl-rambo and Drob-1/Debcl induced the release of cytochrome *c* into the cytosol. These results suggest that Bcl-rambo regulates the mitochondrial signaling pathway of apoptosis.

Human Bcl-2 and murine Bax have been shown to play roles in the regulation of apoptosis in the *Drosophila* eye and wing [48]. Human Bcl-2 is known to suppress murine Bax- and *reaper*-induced mitochondrial defects [49]. Furthermore, Drob-1/Debcl is required for cell death by murine Bax in the *Drosophila* eye [50]. These findings collectively suggest that the apoptosis pathway regulated by Bcl-2 family proteins is highly conserved between mammals and *Drosophila*. We showed that Bcl-rambo did not interact with anti-apoptotic or pro-apoptotic Bcl-2 family members in HEK293T cells [12]. Consistent with this result, Bcl-rambo interacted genetically with Drob-1/Debcl or Buffy only weakly in *Drosophila*. In addition, Bcl-rambo did not bind to Drob-1/Debcl or Buffy in HEK293T cells. Since the BHNo domain of Bcl-rambo was previously shown to induce caspase activation in HEK293T cells [12], the BHNo domain may be essential for the induction of apoptosis in *Drosophila*.

Bcl-rambo has been reported to regulate apoptosis by interacting with adenine nucleotide translocators [21], ceramide synthases 2 (CerS2) and 6 (CerS6) [17] and the *Legionella* protein SidF [20]. Adenine nucleotide translocators are components of the permeability transition pore complex in mitochondria, and play a key role in cell death [51]. In *Drosophila*, adenine nucleotide translocators are encoded by *sesB* as a major isoform and the second isogene *Ant2* [52]. Ceramide synthases have been shown to regulate sphingolipid metabolism and also play a role in regulating cell death [53,54]. In subcellular localization, ceramide synthase activities have been detected in mitochondrial and microsomal fractions [54]. Schlank is the *Drosophila* member of the ceramide synthase family and controls growth and body fat [55]. Due to its localization to mitochondria, it was possible to postulate that Bcl-rambo may regulate the mitochondrial apoptosis pathway via adenine nucleotide translocators or ceramide synthases. The genetic interaction between Bcl-rambo and SesB, but not Schlank suggests that Bcl-rambo regulates the mitochondrial apoptosis pathway primarily via adenine nucleotide transporters in *Drosophila*.

The baculovirus p35 and *Drosophila* Diap1 and Diap2 have been shown to inhibit caspases in order to prevent apoptosis [28,42]. Bcl-rambo induced caspase activity in the eye imaginal discs of third instar larvae, but not the pupal retinae, suggesting that Bcl-rambo has a stronger effect on dividing cells. The ectopic expression of Bcl-rambo induced the rough eye phenotype in *Drosophila*, accompanied by a reduction in eye size and the loss of eye pigment. Although the reduced eye size caused by Bcl-rambo was only weakly rescued by p35, Diap1, and Diap2, reduced pigmentation was rescued by p35, Diap1 and Diap2. The shapes of cone cells and pigment cells were markedly altered by Bcl-rambo, and their morphologies were also partially rescued by p35 in the pupal retinae. Thus, the overexpression of Bcl-rambo may have multiple effects on cells, and the loss of cells may be secondary to defects rescued by p35. Previous studies reported that the ectopic expression of Drob-1/Debcl resulted in the rough eye phenotype [33,34], while the rough eye phenotype induced by Drob-1/Debcl was inhibited by p35 [33]. Drob-1/Debcl-induced cell death was not antagonized by p35 in *Drosophila* S2 cells [34], while it was rescued by p35 in CHO cells [32]. These findings suggest that the role of p35 in Drob-1/Debcl-induced cell death may be influenced by the cellular context. In addition to apoptosis, necrosis and autophagic cell death have been observed during *Drosophila* development [56,57]. Drob-1/Debcl and Buffy are associated with germinal cell death characterized with the mixed morphologies of apoptosis and necrosis [58]. Thus, the ectopic expression of Bcl-rambo may induce caspase-dependent apoptosis and caspase-independent cell death in *Drosophila*.

In conclusion, the ectopic expression of Bcl-rambo induced the rough eye phenotype in transgenic flies. Bcl-rambo induced the rough eye phenotype, which suggested that a functional homologue of Bcl-rambo may exist in *Drosophila* even though amino acid homology is small. A recent study showed that Bcl-rambo had a short WXXL/I motif and was a mammalian homologue of yeast Atg32 that mediated mitophagy and mitochondrial fragmentation [23]. Bcl-rambo has been reported to interact with adenine nucleotide translocators, ceramide synthases, and LC3 (a mammalian homologue of Atg8) [17,21,23], and homologues of these proteins are present in *Drosophila*. Thus, humans and *Drosophila* may have a similar pathway regulated by Bcl-rambo. By using *Drosophila* as a screening system, we revealed that human Bcl-rambo interacted genetically with *Drosophila* homologues of adenine nucleotide translocators and Atg8, suggesting that Bcl-rambo mediates at least two different pathways for apoptosis and mitophagy in *Drosophila*. These results indicate that the *Drosophila* model established in the present study can be a powerful screening tool for investigating genetic interactions. The further identification of gene(s) interacting with Bcl-rambo may help to address its physiological function in humans.

## Supporting Information

S1 Fig Bcl-rambo lacking the C-terminal TM did not localize to mitochondria in *Drosophila* S2 cells. (A) Structures of human Bcl-rambo and its mutants. (B) S2 cells were transfected with pMT-V5-His A, pMT-V5-His A/*Bcl-rambo (1–441)*, or pMT-V5-His A/*Bcl-rambo (1–459)* and then incubated in the presence of CuSO_4_ (500 μM) and Z-VAD-fmk (20 μM) for 24 h. S2 cells were stained for Bcl-rambo (green) and with DAPI (blue) and MitoTracker^®^ Red (red). The stained cells in at least five different fields were observed by confocal laser scanning microscopy. Optical sections containing a single transfected cell are shown. Data were representative of two independent experiments. Scale bars indicate 10 μm.
(TIF)

S2 Fig Bcl-rambo lacking the C-terminal TM did not induce apoptosis in *Drosophila* S2 cells. (A) S2 cells were transfected with pAct5C-*GAL4* together with pMT-V5-His A, pMT-V5-His A/*Bcl-rambo*, pMT-V5-His A/*Bcl-rambo (ΔTM)*, pMT-V5-His A/*Bcl-rambo (1–441)*, pMT-V5-His A/*Bcl-rambo (1–459)*, or pUAST-*DsRed-monomer* in the presence of CuSO_4_ (500 μM) for 24 h. Cells were stained with Hoechst 33342. Nuclear morphology was observed by fluorescent microscopy. Apoptotic cells (%) are shown as the mean ± S.E. of three independent experiments. **P<0.01, significantly different from the control. n.s., not significant. Transfection efficiency was measured by counting DsRed monomer-expressing cells, and calculated to be 16.5 ± 0.4% (the mean ± S.E of three independent experiments).
(TIF)

S3 Fig Ectopic expression of Bcl-rambo, but not Bcl-rambo (ΔTM) induced a rough eye phenotype in three additional fly lines. (A and B) Bcl-rambo and Bcl-rambo (ΔTM) were expressed using *GMR-GAL4* driver fly lines. (A) (a, d, g) *GMR-GAL4*/*w*; +; *UAS-Bcl-rambo*/+ (strain #1), (b, e, h) *GMR-GAL4*/*UAS-Bcl-rambo*; +; + (strain #6), (c, f, i) *GMR-GAL4*/*w*; *UAS-Bcl-rambo*/+; + (strain #46) (B) (a, d, g) *GMR-GAL4*/*w*; *UAS-Bcl-rambo* (ΔTM)/+; + (strain #10), (b, e, h) *GMR-GAL4*/*w*; *UAS-Bcl-rambo* (ΔTM)/+; + (strain #26), (c, f, i) *GMR-GAL4*/*UAS-Bcl-rambo* (ΔTM); +; + (strain #148) The morphology of adult eyes was observed by light microscopy (a–c) and SEM (d–i). Scale bars in d–f and g–i indicate 50 μm and 14.2 μm, respectively. Data were representative of two independent experiments.
(TIF)

S4 Fig Additional UAS repeats in the *UAS*-*GFP* insertion did not rescue the rough eye phenotype induced by Bcl-rambo. (A and B) Bcl-rambo and Bcl-rambo (ΔTM) were expressed using *GMR*-*GAL4* driver fly lines. (A) (a, e, i) *GMR-GAL4*/+; *UAS-Bcl-rambo*/*UAS-GFP*; +, (b, f, j) *GMR-GAL4*/*w*; *UAS-Bcl-rambo*/+; *UAS-p35*/+, (c, g, k) *GMR-GAL4*/*w*; *UAS-Bcl-rambo*/+; *UAS-Diap1*/+, (d, h, l) *GMR-GAL4/w*; *UAS-Bcl-rambo/+*; *GMR-Diap2/+*. (B) (a, e, i) *GMR-GAL4/+*; *UAS-Bcl-rambo (ΔTM)/UAS-GFP*; +, (b, f, j) *GMR-GAL4/w*; *UAS-Bcl-rambo (ΔTM)/+*; *UAS-p35/+*, (c, g, k) *GMR-GAL4/w*; *UAS-Bcl-rambo (ΔTM)/+*; *UAS-Diap1/+*, (d, h, l) *GMR-GAL4/w*; *UAS-Bcl-rambo (ΔTM)/+*; *GMR-Diap2/+*. The morphology of adult eyes was observed by light microscopy (a–d) and SEM (e–l). Scale bars in e–h and i–l indicate 50 μm and 14.2 μm, respectively. Data were representative of two independent experiments.
(TIF)

S5 Fig Bcl-rambo did not interact genetically with Buffy. Bcl-rambo and Bcl-rambo (ΔTM) were expressed using *GMR*-*GAL4* driver fly lines. (A) (a, e, i) *GMR-GAL4*/*w*; *UAS-Bcl-rambo*/+; +, (b, f, j) *GMR-GAL4/w*; *UAS-Bcl-rambo/Buffy*^*H37*^; +, (c, g, k) *GMR-GAL4/w*; *UAS-Bcl-rambo (ΔTM)/+*; +, (d, h, l) *GMR-GAL4/w*; *UAS-Bcl-rambo (ΔTM)/ Buffy*^*H37*^; +. The morphology of adult eyes was observed by light microscopy (a–d) and SEM (e–l). Scale bars in e–h and i–l indicate 50 μm and 14.2 μm, respectively. Data were representative of two independent experiments.
(TIF)
