# Supplementary figures and images for: The Human Bcl-2 Family Member Bcl-rambo Localizes to Mitochondria and Induces Apoptosis and Morphological Aberrations in Drosophila
Source: PLoS One. 2016 Jun 27;11(6):e0157823. doi: 10.1371/journal.pone.0157823 (PMC4922555; doi:10.1371/journal.pone.0157823)

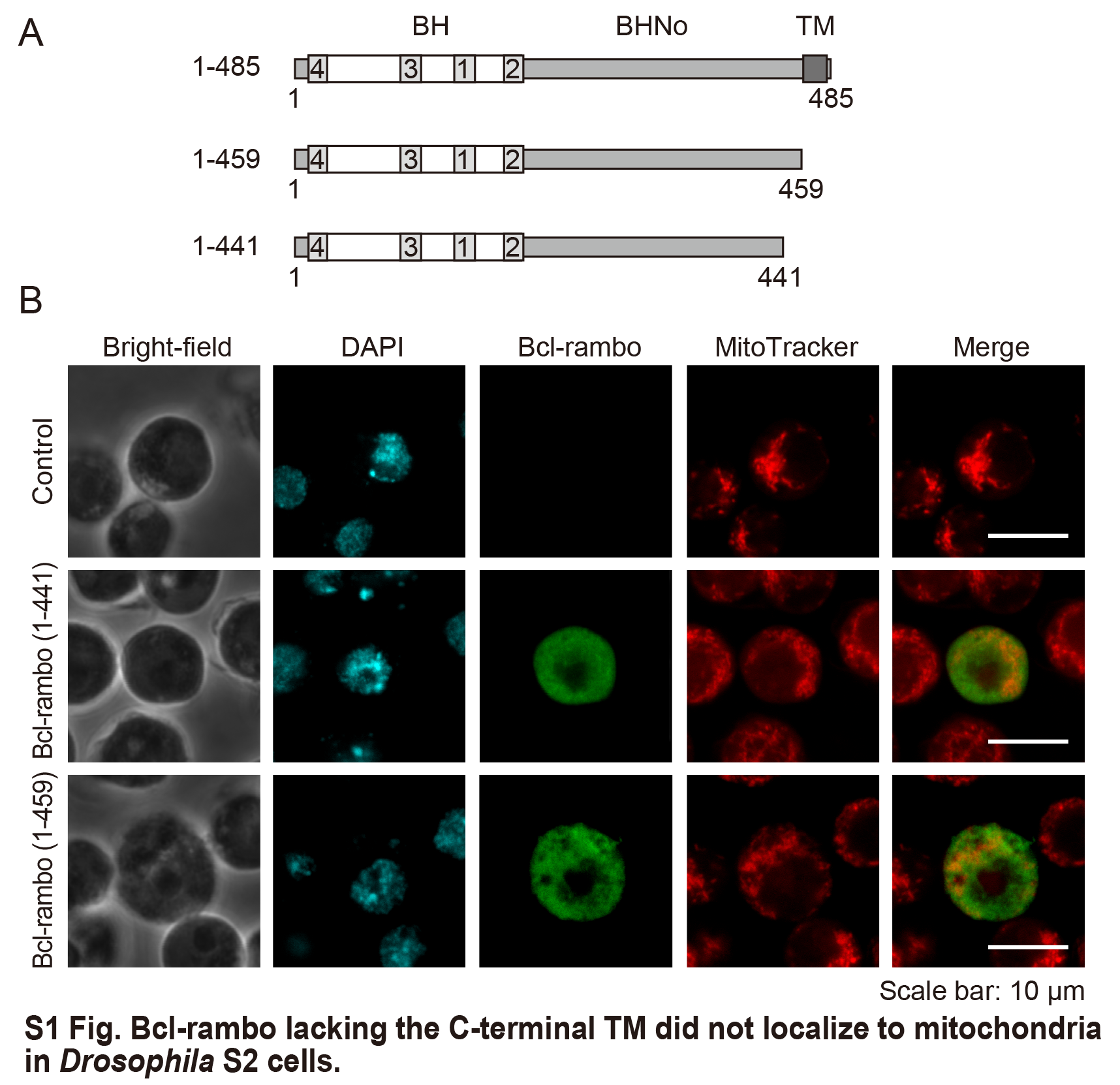

Supplement: S1 Fig — (A) Structures of human Bcl-rambo and its mutants. (B) S2 cells were transfected with pMT-V5-His A, pMT-V5-His A/Bcl-rambo (1–441), or pMT-V5-His A/Bcl-rambo (1–459) and then incubated in the presence of CuSO4 (500 μM) and Z-VAD-fmk (20 μM) for 24 h. S2 cells were stained for Bcl-rambo (green) and with DAPI (blue) and MitoTracker® Red (red). The stained cells in at least five different fields were observed by confocal laser scanning microscopy. Optical sections containing a single transfected cell are shown. Data were representative of two independent experiments. Scale bars indicate 10 μm. (TIF) [file pone.0157823.s001.tif]

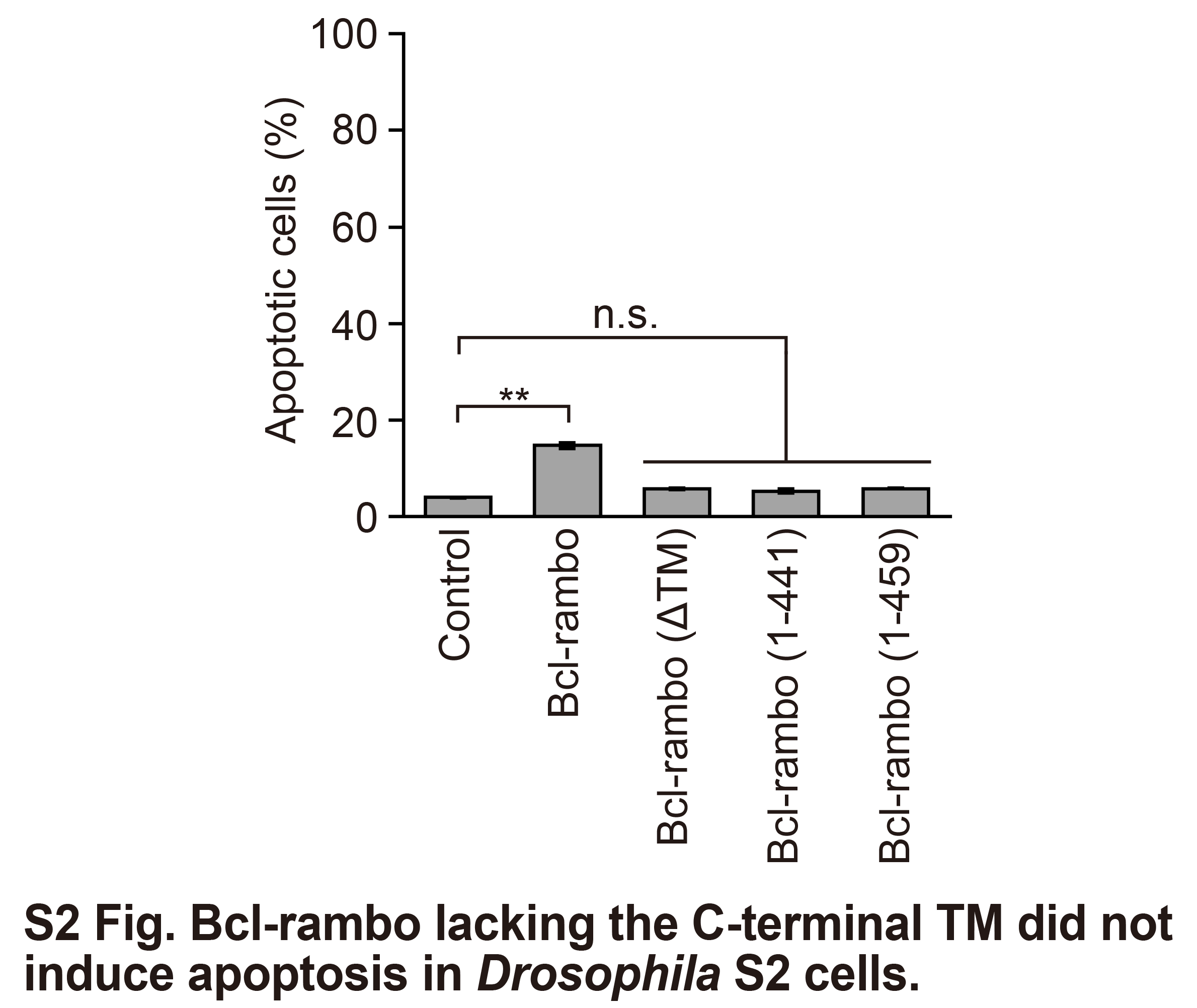

Supplement: S2 Fig — (A) S2 cells were transfected with pAct5C-GAL4 together with pMT-V5-His A, pMT-V5-His A/Bcl-rambo, pMT-V5-His A/Bcl-rambo (ΔTM), pMT-V5-His A/Bcl-rambo (1–441), pMT-V5-His A/Bcl-rambo (1–459), or pUAST-DsRed-monomer in the presence of CuSO4 (500 μM) for 24 h. Cells were stained with Hoechst 33342. Nuclear morphology was observed by fluorescent microscopy. Apoptotic cells (%) are shown as the mean ± S.E. of three independent experiments. **P<0.01, significantly different from the control. n.s., not significant. Transfection efficiency was measured by counting DsRed monomer-expressing cells, and calculated to be 16.5 ± 0.4% (the mean ± S.E of three independent experiments). (TIF) [file pone.0157823.s002.tif]

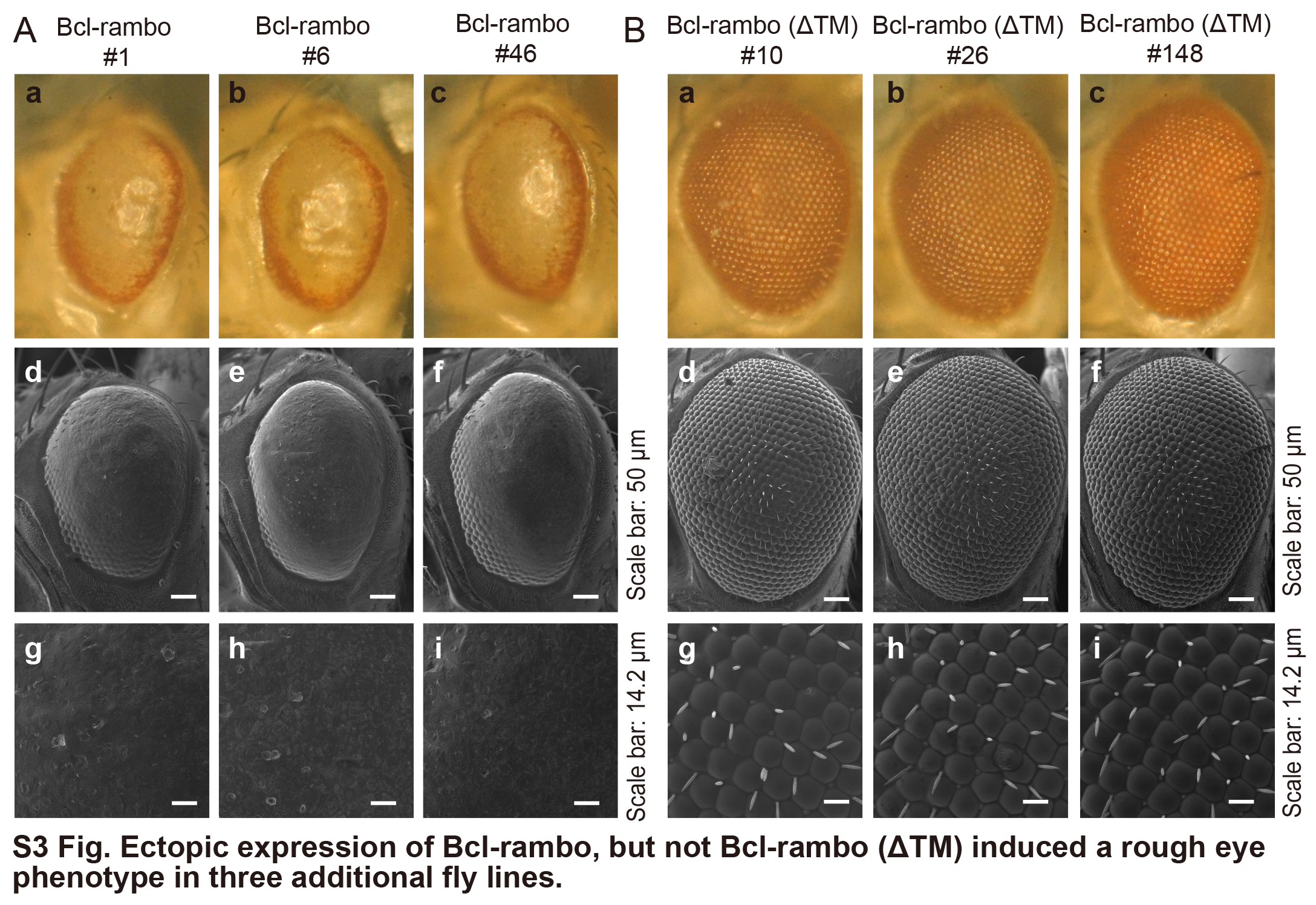

Supplement: S3 Fig — (A and B) Bcl-rambo and Bcl-rambo (ΔTM) were expressed using GMR-GAL4 driver fly lines. (A) (a, d, g) GMR-GAL4/w; +; UAS-Bcl-rambo/+ (strain #1), (b, e, h) GMR-GAL4/UAS-Bcl-rambo; +; + (strain #6), (c, f, i) GMR-GAL4/w; UAS-Bcl-rambo/+; + (strain #46) (B) (a, d, g) GMR-GAL4/w; UAS-Bcl-rambo (ΔTM)/+; + (strain #10), (b, e, h) GMR-GAL4/w; UAS-Bcl-rambo (ΔTM)/+; + (strain #26), (c, f, i) GMR-GAL4/UAS-Bcl-rambo (ΔTM); +; + (strain #148) The morphology of adult eyes was observed by light microscopy (a–c) and SEM (d–i). Scale bars in d–f and g–i indicate 50 μm and 14.2 μm, respectively. Data were representative of two independent experiments. (TIF) [file pone.0157823.s003.tif]

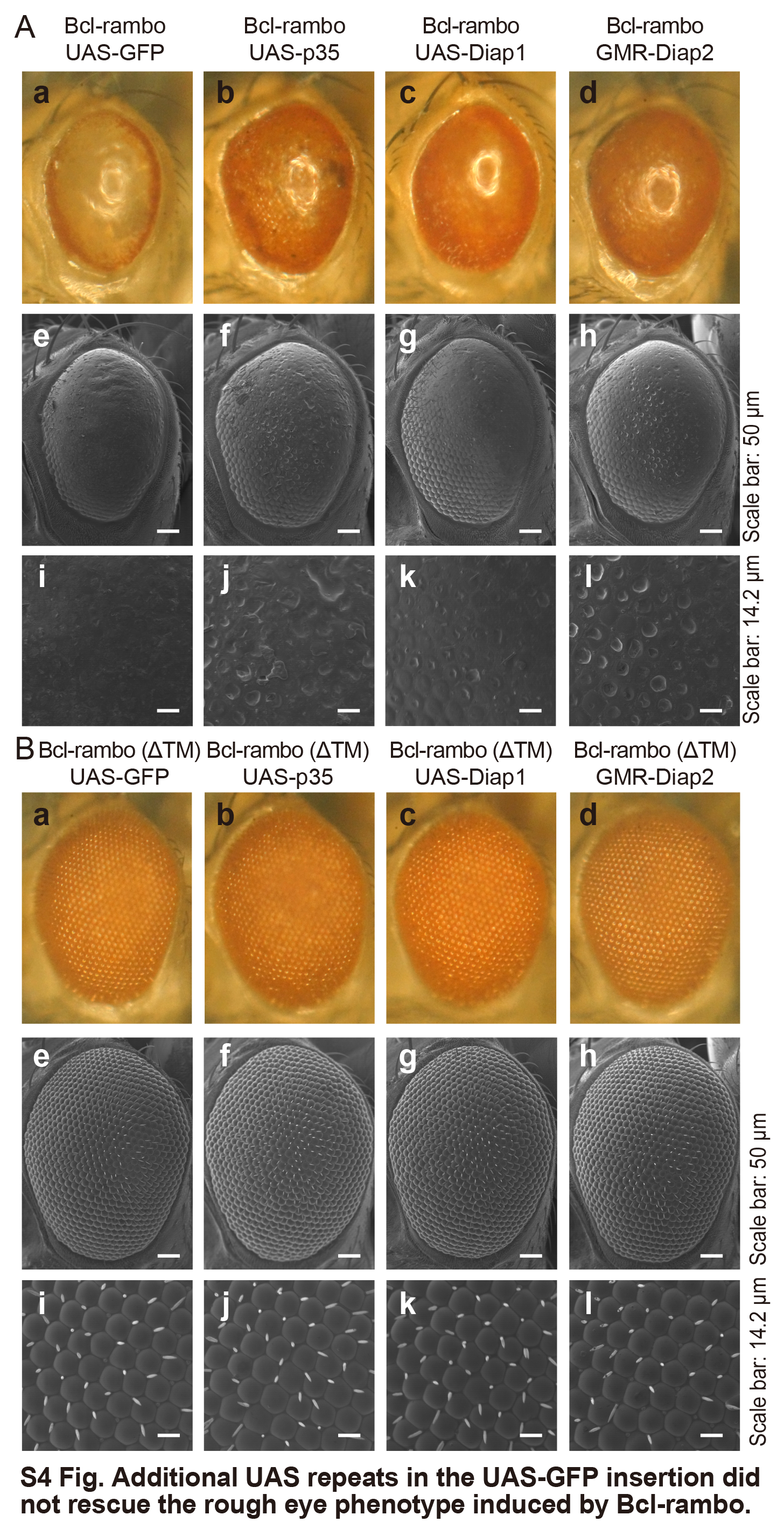

Supplement: S4 Fig — (A and B) Bcl-rambo and Bcl-rambo (ΔTM) were expressed using GMR-GAL4 driver fly lines. (A) (a, e, i) GMR-GAL4/+; UAS-Bcl-rambo/UAS-GFP; +, (b, f, j) GMR-GAL4/w; UAS-Bcl-rambo/+; UAS-p35/+, (c, g, k) GMR-GAL4/w; UAS-Bcl-rambo/+; UAS-Diap1/+, (d, h, l) GMR-GAL4/w; UAS-Bcl-rambo/+; GMR-Diap2/+. (B) (a, e, i) GMR-GAL4/+; UAS-Bcl-rambo (ΔTM)/UAS-GFP; +, (b, f, j) GMR-GAL4/w; UAS-Bcl-rambo (ΔTM)/+; UAS-p35/+, (c, g, k) GMR-GAL4/w; UAS-Bcl-rambo (ΔTM)/+; UAS-Diap1/+, (d, h, l) GMR-GAL4/w; UAS-Bcl-rambo (ΔTM)/+; GMR-Diap2/+. The morphology of adult eyes was observed by light microscopy (a–d) and SEM (e–l). Scale bars in e–h and i–l indicate 50 μm and 14.2 μm, respectively. Data were representative of two independent experiments. (TIF) [file pone.0157823.s004.tif]

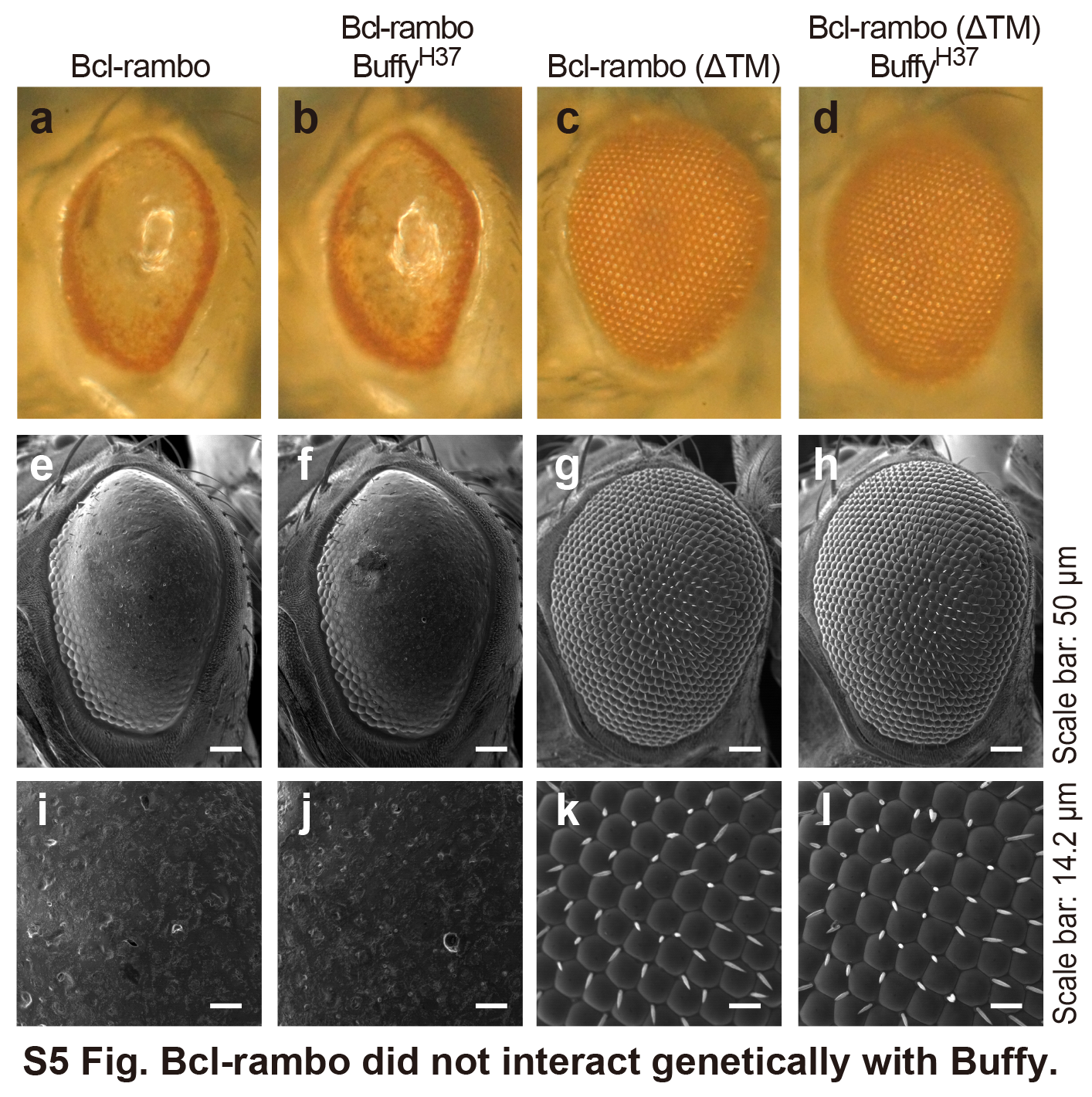

Supplement: S5 Fig — Bcl-rambo and Bcl-rambo (ΔTM) were expressed using GMR-GAL4 driver fly lines. (A) (a, e, i) GMR-GAL4/w; UAS-Bcl-rambo/+; +, (b, f, j) GMR-GAL4/w; UAS-Bcl-rambo/BuffyH37; +, (c, g, k) GMR-GAL4/w; UAS-Bcl-rambo (ΔTM)/+; +, (d, h, l) GMR-GAL4/w; UAS-Bcl-rambo (ΔTM)/ BuffyH37; +. The morphology of adult eyes was observed by light microscopy (a–d) and SEM (e–l). Scale bars in e–h and i–l indicate 50 μm and 14.2 μm, respectively. Data were representative of two independent experiments. (TIF) [file pone.0157823.s005.tif]
